# HAL-2 Promotes Homologous Pairing during *Caenorhabditis elegans* Meiosis by Antagonizing Inhibitory Effects of Synaptonemal Complex Precursors

**DOI:** 10.1371/journal.pgen.1002880

**Published:** 2012-08-09

**Authors:** Weibin Zhang, Natasha Miley, Michael S. Zastrow, Amy J. MacQueen, Aya Sato, Kentaro Nabeshima, Enrique Martinez-Perez, Susanna Mlynarczyk-Evans, Peter M. Carlton, Anne M. Villeneuve

**Affiliations:** 1 Departments of Developmental Biology and Genetics, Stanford University School of Medicine, Stanford, California, United States of America; 2 Institute for Integrated Cell-Material Sciences (iCeMS), Kyoto University, Yoshida, Sakyo-ku, Kyoto, Japan; Stowers Institute for Medical Research, United States of America

## Abstract

During meiosis, chromosomes align with their homologous pairing partners and stabilize this alignment through assembly of the synaptonemal complex (SC). Since the SC assembles cooperatively yet is indifferent to homology, pairing and SC assembly must be tightly coordinated. We identify HAL-2 as a key mediator in this coordination, showing that HAL-2 promotes pairing largely by preventing detrimental effects of SC precursors (SYP proteins). *hal-2* mutants fail to establish pairing and lack multiple markers of chromosome movement mediated by pairing centers (PCs), chromosome sites that link chromosomes to cytoplasmic microtubules through nuclear envelope-spanning complexes. Moreover, SYP proteins load inappropriately along individual unpaired chromosomes in *hal-2* mutants, and markers of PC-dependent movement and function are restored in *hal-2; syp* double mutants. These and other data indicate that SYP proteins can impede pairing and that HAL-2 promotes pairing predominantly but not exclusively by counteracting this inhibition, thereby enabling activation and regulation of PC function. HAL-2 concentrates in the germ cell nucleoplasm and colocalizes with SYP proteins in nuclear aggregates when SC assembly is prevented. We propose that HAL-2 functions to shepherd SYP proteins prior to licensing of SC assembly, preventing untimely interactions between SC precursors and chromosomes and allowing sufficient accumulation of precursors for rapid cooperative assembly upon homology verification.

## Author Summary

For successful segregation of homologous chromosomes during sexual reproduction, homologs must first identify and pair with their correct partners. Further, many organisms stabilize and maintain alignment between paired homologs through assembly of a highly ordered structure known as the synaptonemal complex (SC). Pairing and synapsis must be tightly coordinated to ensure that SC assembly only occurs in a productive manner, linking the axes of correctly aligned homologous chromosomes. In this work, we identify HAL-2, a protein that concentrates in the nucleoplasm of germ cells, as a key player in mediating this coordination. We find that precursors of the SC have the potential to inhibit homolog pairing, interfering with the very process that the SC normally serves to stabilize. Moreover, we show that HAL-2 promotes homolog pairing and associated chromosome movement primarily by counteracting these detrimental inhibitory effects of SC precursors. Our data suggest that HAL-2 serves to prevent inappropriate association of SC precursors with chromosomes prior to licensing of SC assembly, and we propose that HAL-2 may enable precursors to accumulate in a manner that allows rapid, cooperative SC assembly upon homology verification.

## Introduction

Generation of haploid gametes during sexual reproduction depends critically on the ability of homologous chromosomes to identify and form pairwise associations with their appropriate partners. Pairing is essential to enable homologs to orient and segregate away from each other at the meiosis I division, thereby achieving the reduction in ploidy that is necessary to ensure restoration of the diploid state upon fertilization.

Pairing between homologous chromosomes is reinforced by a highly ordered tripartite structure known as the synaptonemal complex (SC) that assembles at the interface between aligned homologs [1]. The lateral elements (LEs) of the SC are comprised of cohesin complexes and meiosis-specific axial components that coalesce along the length of each homolog during early meiotic prophase. SC central region (CR) proteins, which contain extended coiled-coil domains, then load to create transverse connections between the axes of the paired homologs, conferring the characteristic zipper-like appearance of mature SC. SC proteins collaborate with meiosis-specific recombination proteins to promote crossover recombination events between the homologs and to promote maturation of chromosome structure surrounding crossover sites into temporary connections known as chiasmata that enable homolog segregation at the meiosis I division [2].

Although the SC normally assembles only between paired homologous chromosomes, the SC structure itself is indifferent to homology. Moreover, SC assembly is highly cooperative and processive, such that once synapsis is initiated, it can proceed along nonhomologous chromosome segments [1]. Further, CR proteins have a propensity to self-assemble into aggregates known as polycomplexes even when they are not associated with chromosomes [3]–[6]. Thus, homolog pairing and synapsis must be tightly coordinated to ensure that SC assembly only occurs in a productive fashion, linking the axes of correctly aligned homologs.

During *Caenorhabditis elegans* meiosis, much of the coordination between pairing and synapsis is mediated by specialized chromosomal domains termed pairing centers (PCs) that are located near one end of each chromosome [7]–[9]. PCs function both in conferring SC-independent local stabilization of pairing and in promoting SC assembly [8], [10]. A family of zinc finger proteins (HIM-8, ZIM-1, ZIM-2 and ZIM-3) that bind to specific PCs mediates functional connections between chromosomes and cytoplasmic dynein and microtubules through a conserved nuclear envelope-spanning complex (which includes KASH-domain protein ZYG-12 and SUN-domain protein SUN-1) that promotes dramatic intranuclear chromosome movements [11]–[17]. In addition to facilitating timely pairing between homologs, these movements also appear to play a role in licensing SC assembly [14]. Dynein-dependent forces acting at PCs have been proposed to function in a checkpoint-like mechanism that makes SC assembly contingent upon homology verification [14], [18]. Both the ZYG-12/SUN-1 complex and the HORMA domain LE component HTP-1 have been implicated in the operation of this proposed coupling mechanism, serving to impose a barrier to SC assembly that can be overcome by interactions between homologous PCs, thereby preventing SC assembly between nonhomologous chromosomes [14], [16], [18]. Further, HTP-1 has also been implicated in a second coordination mechanism that couples termination of chromosome movement with SC installation [18].

Here we identify a nucleoplasmic component of the meiotic machinery, HAL-2 *(homolog alignment-2)*, that provides a distinct contribution to the coordination of homolog pairing and synapsis. We find that HAL-2 promotes pairing between homologous chromosomes predominantly by counteracting detrimental inhibitory effects of CR precursors (SYP proteins). By preventing uncontrolled behavior of SYP proteins that interferes with PC function and antagonizes HTP-1/2 localization, HAL-2 enables chromosome movement and homology assessment. Additionally, we find that HAL-2 also has SYP-independent roles in regulating progression of meiotic prophase events, by contributing to the coupling mechanism that maintains chromosome mobilization in response to unsynapsed chromosomes and by promoting initiation of recombination.

## Results

### *hal-2* mutants are defective in nuclear reorganization and homolog pairing during meiotic prophase

**Figure 1:**
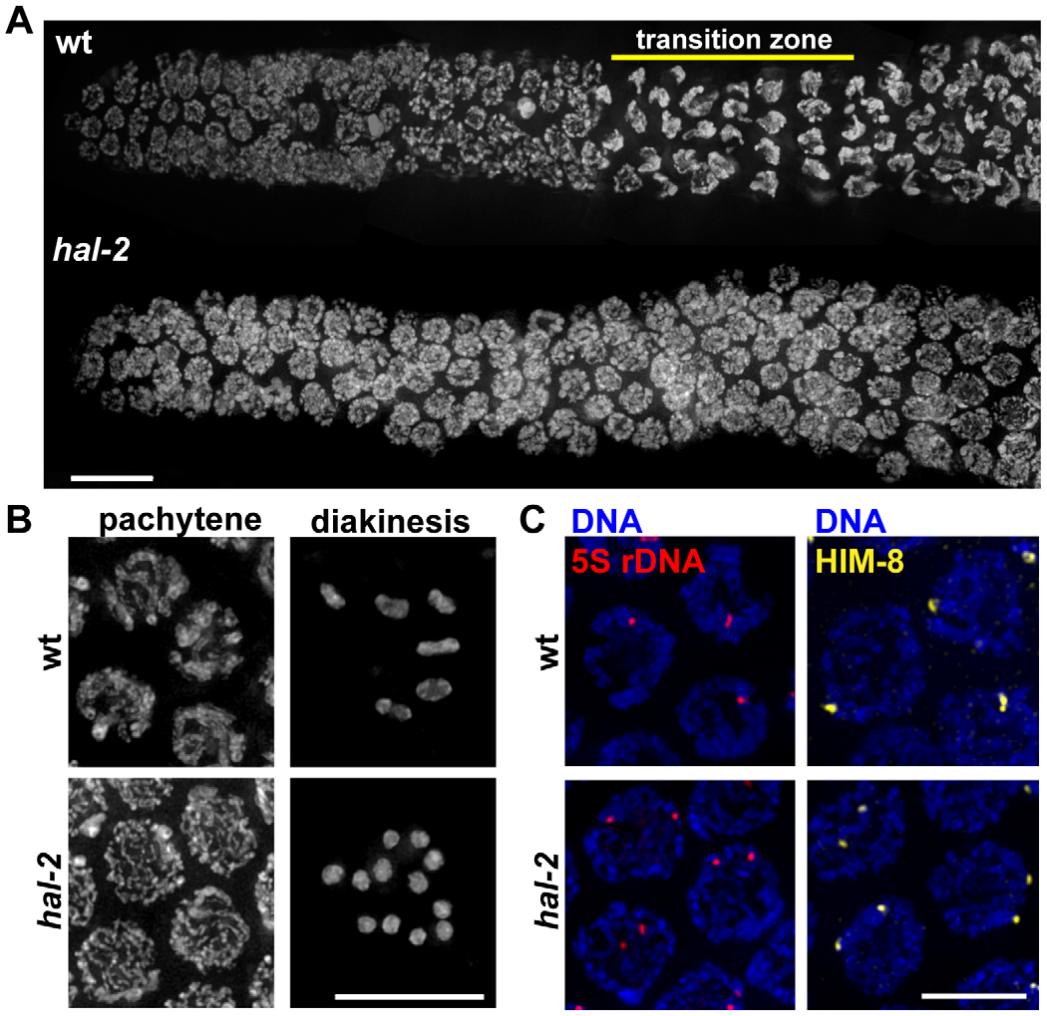
Defective nuclear reorganization and homologous pairing in *hal-2* mutants. (A) Chromosome organization in germline nuclei in regions of wild-type (wt) and *hal-2* mutant gonads extending from the premeiotic zone (left) through early pachytene (right). In the wild-type gonad, DAPI-stained chromatin appears widely dispersed in premeiotic nuclei and exhibits a highly clustered organization in nuclei within the transition zone (TZ), reflecting clustering of chromosomes during a period of active chromosome mobilization that begins soon after meiotic entry; DAPI signals appear more dispersed in nuclei that have exited the TZ and progressed into pachytene. The *hal-2* mutant gonad lacks nuclei with the clustered chromosome configuration characteristic of the TZ, indicating impairment of nuclear reorganization upon meiotic entry. Bar, 10 µm. (B) Left: Wild-type and *hal-2* mutant nuclei at mid-pachytene. The wild-type nuclei contain thick parallel tracks of DAPI-stained chromatin corresponding to aligned homolog pairs, whereas the *hal-2* mutant nuclei contain disorganized thin DAPI-stained chromatin tracks representing unaligned chromosomes. Right: Each panel shows DAPI-stained chromosomes in a single oocyte at diakinesis, the last stage of meiotic prophase. Whereas 6 bivalents are present in the wild-type oocyte, each representing a homolog pair held together by a chiasma, 12 smaller DAPI-stained bodies (univalents) are observed in the *hal-2* mutant oocyte, indicating an absence of chiasmata. Bar, 10 µm. (C) 5S rDNA FISH (left) and immunofluorescence (IF) of X chromosome PC-binding protein HIM-8 (right) in pachtyene nuclei from wild-type and *hal-2* germ lines. A single focus or two closely spaced foci is detected in wild-type nuclei, indicating paired homologs. Two widely spaced foci are seen in the *hal-2* mutant nuclei, indicating that both the X-PC and 5S rDNA locus were unpaired. Bar, 5 µm.

An essential role for HAL-2 in promoting nuclear reorganization and homolog pairing was revealed through analysis of the *hal-2(me79)* mutant, which was isolated in a screen for mutants with defective meiotic chromosome segregation [19], [20]. Cytological analysis of the *hal-2(me79)* mutant revealed defects in chromosome organization during meiotic prophase. In wild-type germ cell nuclei entering meiotic prophase (in a region of the gonad called the transition zone, TZ), chromosomes are dramatically reorganized into a clustered configuration that reflects active chromosome movement (Figure 1A) [14], [15], [21], [22]. In the *hal-2* mutant, germ cell nuclei entering meiotic prophase lack this characteristic TZ organization and chromosomes are instead dispersed around the nuclear periphery (Figure 1A). Further, whereas wild-type nuclei at the mid-pachytene stage exhibit parallel tracks of DAPI-stained DNA corresponding to aligned homolog pairs, *hal-2* mutant nuclei at this stage exhibit disorganized single DAPI tracks (Figure 1B). This organization reflects a severe impairment of homolog pairing in the *hal-2* mutant, as assessed at the 5S locus on chromosome V (by fluorescence *in situ* hybridization [FISH]) and at the X chromosome PC (X-PC) (by immunofluorescence [IF] for X-PC binding protein HIM-8) (Figure 1C). In both assays, the *hal-2* mutant exhibited two widely separated foci in most nuclei (see quantitation below), indicating a failure to achieve homolog pairing.

As expected given the pairing defect, diakinesis oocytes in the *hal-2* mutant contain 12 univalents, indicating a lack of chiasmata connecting homologous chromosomes (Figure 1B). Absence of chiasmata results in impaired meiotic chromosome segregation, with *hal-2* mutant hermaphrodites producing both a high frequency of inviable embryos (87.2%, *n* = 1147), indicative of autosomal aneuploidy, and a high incidence of males (Him) among the surviving progeny, indicative of X chromosome missegregation (32.7%, *n* = 147) (Table S1) [23].

### *hal-2* mutants lack multiple markers of PC-mediated chromosomal movement

**Figure 2:**
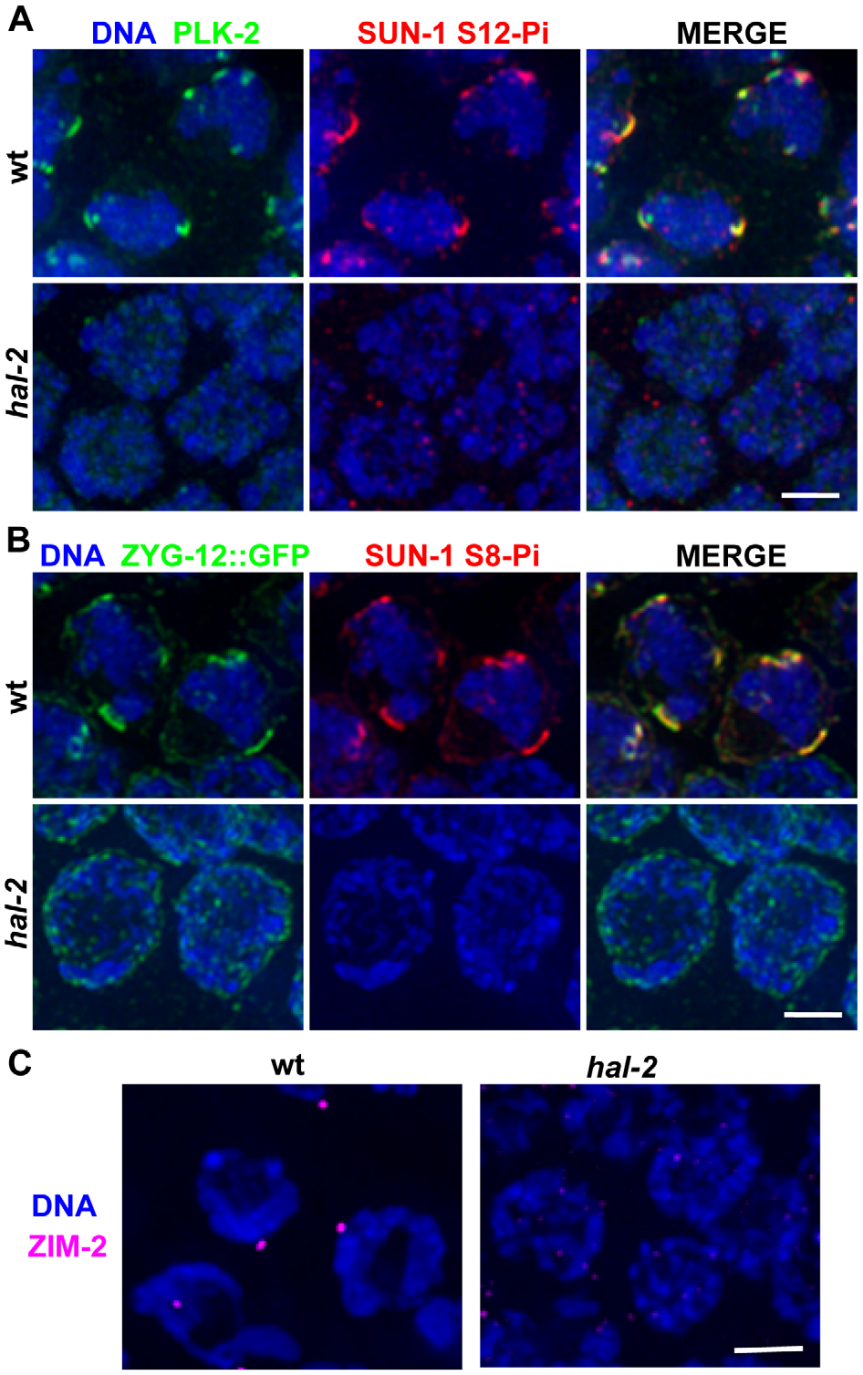
*hal-2* mutants lack multiple markers of PC-mediated chromosomal movement. (A) IF images of early prophase nuclei stained for PLK-2 and SUN-1 phosphorylated on Ser12 (SUN-1 S12-Pi) in wild-type and *hal-2* animals. In the wild-type nuclei, SUN-1 S12-Pi and PLK-2 are concentrated together in bright patches at the nuclear envelope (NE). In the *hal-2* nuclei, PLK-2 and SUN-1 S12-Pi are not detected. Bar, 2 µm. (B) IF images of early prophase nuclei stained for ZYG-12::GFP and SUN-1 phosphorylated on Ser8 (SUN-1 S8-Pi) in wild-type and *hal-2* animals carrying the *zyg-12::gfp* transgene. In the wild-type nuclei, SUN-1 S8-Pi and ZYG-12::GFP are concentrated together in bright patches at the NE and are also detected at lower levels throughout the entire NE. In the *hal-2* nuclei shown, SUN-1 S8-Pi is not detected and ZYG-12::GFP is dispersed throughout the NE. Bar, 2 µm. (C) IF images of ZIM-2 (chromosome V PC-binding protein) in early prophase nuclei. A single bright focus near the NE is observed in the wild-type nuclei, whereas none is detected in the *hal-2* mutant nuclei. Bar, 2 µm.

Nuclear reorganization and initiation of homologous pairing in the TZs of wild-type worms are accompanied by dramatic chromosomal movements that are mediated by association of PCs and their binding proteins with patches of nuclear envelope (NE) proteins that are in turn connected to cytoplasmic microtubules [14]–[16]. SUN-1, an inner NE protein, is phosphorylated on multiple residues upon meiotic entry in a CHK-2-dependent fashion, and together with the outer NE protein ZYG-12, is reorganized into NE patches in wild-type TZ nuclei [11], [14]–[17]. Further, polo-like kinase PLK-2, which promotes phosphorylation of Ser12 (S12-Pi) of SUN-1, colocalizes during early meiotic prophase with PCs, ZYG-12 and phosphorylated SUN-1 in these NE patches and promotes their formation [24], [25]. IF analysis revealed that PLK-2 is not localized to NE patches in early prophase nuclei in *hal-2* mutants, and SUN-1 S12-Pi is not detected (Figure 2A). Further, even though ZYG-12::GFP was localized to the nuclear envelope in the *hal-2* mutant, it was not reorganized into patches, and CHK-2-dependent phosphorylation of Ser8 (S8-Pi) of SUN-1 was severely reduced and/or delayed (Figures 2B and S1). Finally, while live imaging of TZ nuclei in wild-type control germ lines showed chromosome-associated ZYG-12::GFP patches moving rapidly along the nuclear envelope, no evidence of mobile ZYG-12::GFP NE patches was visible in *hal-2* mutant nuclei (Videos S1 and S2). Thus, the modification and reorganization of the NE proteins required for PC-mediated chromosomal movement is severely abrogated in *hal-2* mutants.

We also assessed recruitment of autosomal PC-binding proteins ZIM-2 (chromosome V) and ZIM-3 (chromosomes I and IV) in the *hal-2* mutant. Whereas the X-PC binding protein HIM-8 is associated with the X-PCs and the nuclear envelope throughout most of the germ line, association of the ZIM proteins with their respective PCs and the nuclear envelope occurs most prominently in the TZ during wild-type meiosis, beginning just prior to the onset of homolog pairing [12], [13]. We found that while bright NE-associated HIM-8 foci were detected in *hal-2* mutant nuclei (Figures 1C and S2), no bright ZIM-2 or ZIM-3 foci were detected (Figures 2C and S3A), indicating a failure to concentrate ZIM-2 and ZIM-3 to their respective PCs and the nuclear envelope. In summary, our data indicate that *hal-2* mutants are defective in the assembly of the linkage between PCs and cytoplasmic microtubules (via the NE bridge) required for PC-mediated chromosome movement.

### SYP proteins are loaded onto the axes of unpaired homologs in *hal-2* mutants

**Figure 3:**
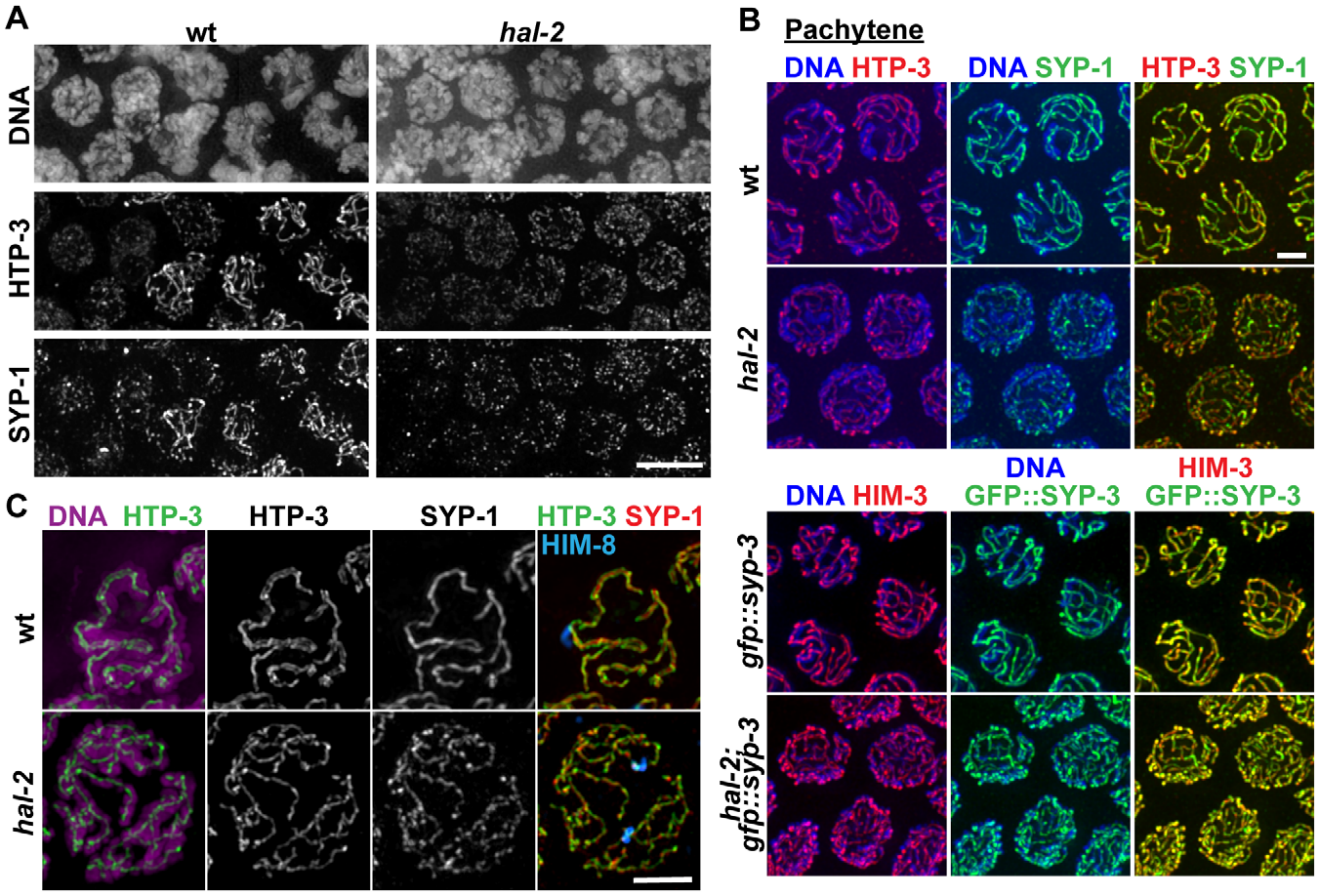
SYP proteins are loaded onto the axes of unpaired homologs in *hal-2* mutants. (A) Portions of wild-type and *hal-2* germ lines extending from premeiotic nuclei (left) to nuclei progressing into meiosis (right), stained with antibodies against HTP-3 (LE component) and SYP-1 (CR protein). In both germ lines, most nuclei in which HTP-3 is detected as discrete axial structures show SYP-1 colocalizing with most HTP-3 stretches, indicating that CR components are competent to load onto the axes of unpaired homologs soon after meiotic entry in the *hal-2* mutant. Bar, 5 µm. (B) Co-immunolocalization of LE components (HTP-3 [top] or HIM-3 [bottom]) and CR proteins (SYP-1 [top] or GFP::SYP-3 [bottom]) in wild-type and *hal-2* nuclei at mid/late pachytene. LE components and SYP proteins colocalize at the interface between DAPI-stained aligned homologs in wild-type nuclei, whereas SYP proteins colocalize with LE components along the lengths of unpaired chromosomes in *hal-2* mutant nuclei. Bar, 2 µm. (C) Localization of LE component HTP-3, CR protein SYP-1 and DAPI-stained chromatin visualized using 3D-SIM; for both genotypes, pairing status of the X chromosomes is indicated by HIM-8 immunostaining (imaged without 3D-SIM). In the wild-type nucleus, two resolvable parallel tracks of HTP-3 flanking a single track of SYP-1 are detected along much of the lengths of the aligned homolog pairs, reflecting the presence of SCs linking pairs of LEs that are separated by a distance of approximately 100 nm [29], [49]. In the *hal-2* nucleus, both SYP-1 and HTP-3 are detected as single tracks at the sister chromatid interface of individual unpaired chromosomes. Bar, 2 µm.

During wild-type meiosis, assembly of the SC between successfully paired homologs is initiated in the TZ. LE components such as HIM-3 and HTP-3 [26], [27] coalesce to form discrete chromosome axes, and these axes are then linked by loading of CR components such as SYP-1 and SYP-3 between them [10], [28], [29]. In standard high-resolution IF images, LE components and SYP proteins colocalize at the interface between aligned homologous chromosomes, beginning in the TZ and during the pachytene stage (Figures 3A and 3B). In meiotic prophase nuclei from similar regions of *hal-2* mutant germ lines, we detected HTP-3 and HIM-3 localized in tracks along the individual chromosomes, indicating that LEs assemble along the unpaired chromosomes in the *hal-2* mutant. Whereas SYP proteins normally load only between paired LEs, however, we found that SYP proteins colocalized with LE components along the lengths of individual unpaired chromosomes in the *hal-2* mutant (Figures 3A and 3B). Colocalization of SYP-1 with HTP-3 in the *hal-2* mutant was detected at about the same time or soon after HTP-3 began to coalesce into discrete axial structures (Figure 3A). These data indicate that in the absence of HAL-2, SYP proteins are able to load onto unpaired chromosome axes.

Images obtained using 3D-structured illumination microscopy (3D-SIM) [30] support this interpretation. In 3D-SIM images of wild-type pachytene nuclei, the two LEs of the aligned homologs (visualized using an α-HTP-3 antibody) can be spatially resolved as two separate entities along much of their lengths, with SYP-1 localized between the two LEs and the associated chromatin of the paired homologs (Figure 3C and Video S3). In 3D-SIM images of *hal-2* mutant pachytene nuclei, SYP-1 was detected mainly in association with single LEs (rather than between resolvable pairs of LEs) along the lengths of the conjoined sister chromatids of unaligned individual chromosomes (Figure 3C and Video S4). Further, in nuclei where short stretches of parallel LE tracks were observed, these corresponded to regions of fold-back near chromosome ends or regions where LEs of two different chromosomes were in close proximity (Figure S4). Thus, the 3D-SIM images are most consistent with SYP proteins loading along individual LEs in the *hal-2* mutant, rather than with formation of SCs between sister chromatid pairs that each had assembled their own LE. Intersister SC formation has been reported to occur in *Rec8* null mutant mice [31] and in the budding yeast *pds5* meiotic-null mutant [32]; in both cases, the widths of the intersister SCs in the mutants were similar to those of interhomolog SCs in wild-type controls [31], [32]. While the lack of extensive resolvable pairs of LEs in our 3D-SIM images of *hal-2* mutant nuclei rules out the formation of intersister SCs of normal width, however, we cannot exclude the possibility that intersister SC-like structures of smaller width might form in *hal-2* mutants.

The loading of SYP proteins onto the axes of unaligned individual chromosomes in *hal-2* mutants contrasts with a distinct class of *C. elegans* pairing mutants (including *htp-1*) in which chromosomes engage in extensive nonhomologous synapsis, with SYP proteins linking the axes of nonhomologous chromosomes [16], [18], [26], [33]. This aspect of the *hal-2* phenotype also contrasts with *chk-2* mutants, in which pairing is abolished and SYP loading is very delayed [18], [20]. We found that *hal-2; htp-1* double mutants load HTP-3 and SYP-1 extensively onto unpaired chromosome axes (as in the *hal-2* single mutant; Figure S5), indicating that nonhomologous synapsis in the *htp-1* mutant is dependent on HAL-2 and suggesting that HAL-2 may be required for SYP proteins to connect chromosome axes together. Likewise, the *hal-2; chk-2* double mutant also resembles the *hal-2* single mutant with respect to loading of HTP-3 and SYP-1 (Figure S5), implying that the ability to inhibit SYP loading on unpaired axes requires HAL-2.

### Removal of SYP proteins in *hal-2* mutants partially restores chromosome clustering, markers of PC-mediated chromosome movement, and homologous pairing

**Figure 4:**
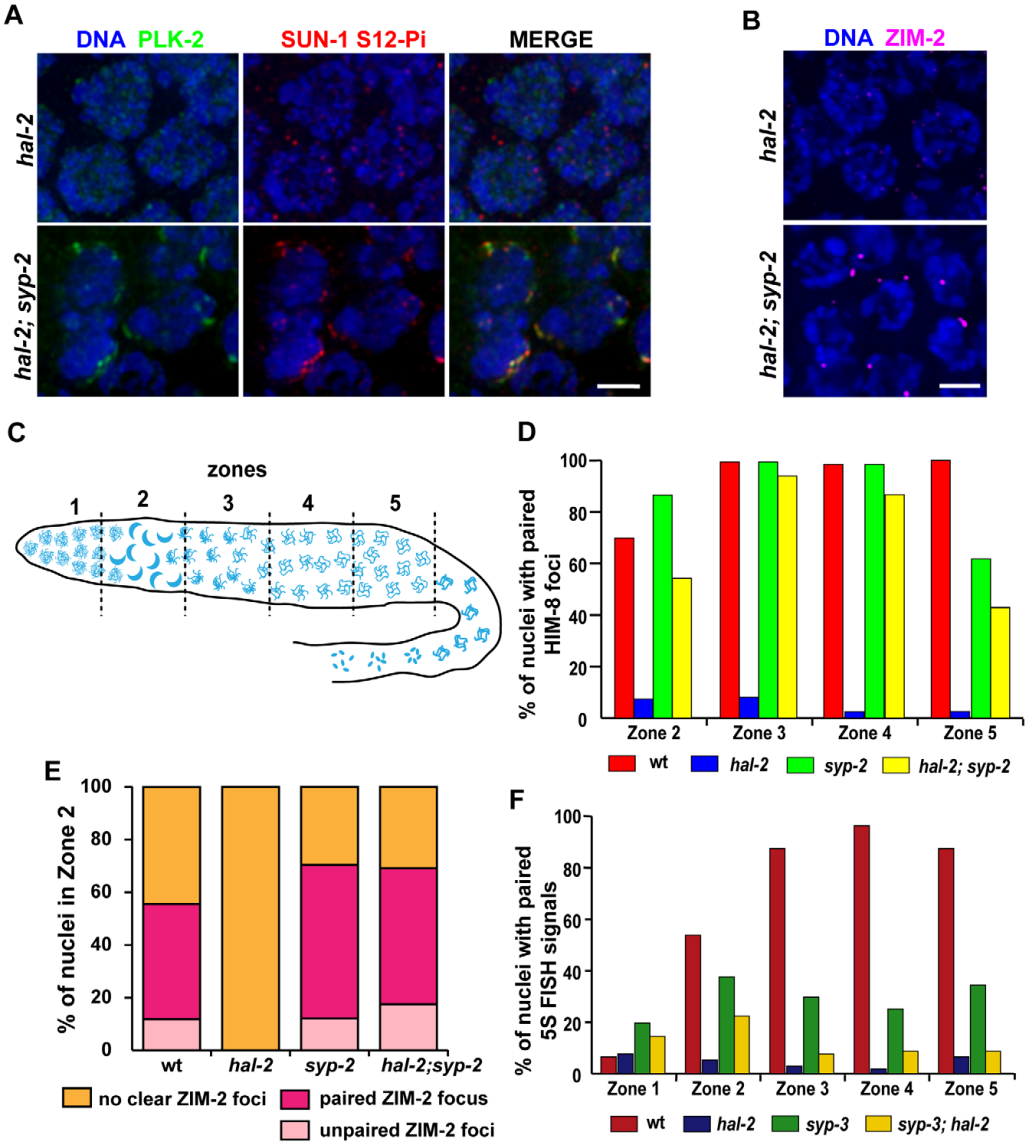
Removal of SYP proteins in *hal-2* mutants partially restores chromosome clustering, markers of PC-mediated chromosome movement, and homologous pairing. (A) IF images of early prophase nuclei stained for PLK-2 and SUN-1 S12-Pi in *hal-2* and *hal-2; syp-2(ok307)* germ lines. Chromosome clustering, SUN-1 Ser12 phosphorylation and NE patches containing PLK-2 and SUN-1 S12-Pi (features that are missing in the *hal-2* nuclei) are restored in the *hal-2; syp-2* nuclei. Bar, 2 µm. (B) *hal-2* and *hal-2; syp-2* early prophase nuclei stained with antibodies against ZIM-2. Whereas ZIM-2 foci are not observed in the *hal-2* mutant nuclei, bright ZIM-2 foci near the NE are restored in the *hal-2; syp-2* double mutants. Bar, 2 µm. (C) Diagram of a hermaphrodite gonad depicting the 5 zones of equal lengths used for quantitation of pairing in Figures 4D–4F. In a wild-type gonad, Zone 1 contains premeiotic nuclei, Zone 2 includes some premeiotic nuclei, the TZ and some early pachytene nuclei, Zone 3 contains early and mid-pachytene nuclei, Zone 4 contains mid and late pachytene nuclei and Zone 5 consists of late pachytene nuclei. (D) Bar graph showing quantitation of pairing at the X-PC, measured as pairing of HIM-8 foci, for Zones 2–5 (the regions with most robust HIM-8 staining). While pairing of HIM-8 foci was abolished in the *hal-2* germ lines, high HIM-8 pairing levels were restored in the *hal-2; syp-2* double mutant, with pairing levels peaking in Zones 3 and 4 (94%; 87%) at levels approaching those of wild-type and the *syp-2* mutant (99%; 98%). Nonetheless pairing levels in *hal-2; syp-2* double mutants were significantly lower than those in *syp-2* gonads for all zones (Zone 2: p<0.0001, Zone 3: p = 0.012, Zone 4: p = 0.0002, Zone 5: p = 0.0194). Worms used in this analysis carried the *zyg-12::gfp* transgene. (E) Quantitation of ZIM-2 foci in nuclei from Zone 2, the region in which ZIM-2 staining is most robust in wild-type germ lines. Stacked bar graphs depict the percentage of Zone 2 nuclei with a single ZIM-2 focus (indicating paired V-PCs), two unpaired ZIM-2 foci (indicating unpaired V-PCs) or without any clear ZIM-2 foci (indicative of inactive PCs not engaged in chromosome mobilization). Worms used in this analysis carried the *zyg-12::gfp* transgene. (F) Bar graph depicting quantitation of pairing levels at the 5S rDNA locus assessed by FISH. A modest partial restoration of pairing at this non-PC locus was observed in the *syp-3(ok758); hal-2* double mutant: 5S rDNA pairing levels were effectively abolished in the *hal-2* mutant, whereas pairing in *syp-3; hal-2* double mutants showed a highly significant improvement over *hal-2* only in Zone 2 (p<0.0001). Further, *syp-3; hal-2* double mutants displayed significantly lower pairing levels than those in *syp-3* gonads for Zones 2–5 (p = 0.0023, p<0.0001, p<0.0001, p<0.0004).

The inappropriate loading of SYP proteins onto unpaired homologs in *hal-2* mutants raised the possibility that the defects related to homologous pairing might be a consequence of the improper association of SYP proteins with chromosomes. To test this hypothesis, we evaluated several features associated with homologous pairing in *hal-2* mutants lacking the SYP proteins. First, we found that removal of SYP proteins (in *hal-2; syp* double mutants) substantially restored multiple features associated with PC-mediated chromosome movement that were eliminated in *hal-2* single mutants. Nuclei with clustered chromosomes and NE patches of PLK-2, ZYG-12::GFP and phosphorylated SUN-1 (S12-Pi and S8-Pi) were detected in *hal-2; syp-2* and/or *syp-3; hal-2* double mutants (Figures 4A, S3B and S6). Further, live imaging of ZYG-12::GFP in *hal-2; syp-2* germ lines indicated that these restored NE patches are competent for movement (Video S5). After the TZ region, *hal-2; syp-2* nuclei also resembled wild-type nuclei in that most nuclei retained a single bright NE focus that was closely associated with HIM-8, indicating that the ZYG-12/SUN-1 NE patch attached to the X chromosomes persists longer than those associated with the autosomes (Figure S7) [14], [15]. In addition, localization of the autosomal PC-binding proteins ZIM-2 and ZIM-3 to bright foci at the nuclear periphery in nuclei with clustered chromosomes was also observed in *hal-2; syp-2* double mutants (Figures 4B and S3A). Finally, restoration of chromosome clustering and SUN-1 S8-Pi in the *syp-3; hal-2* mutants is dependent on CHK-2, as these features were absent in *syp-3; hal-2; chk-2* triple mutants (Figure S6). Taken together, these findings indicate that CHK-2-/PLK-2-dependent PC-mediated chromosome mobilization can be inhibited by SYP proteins, and that HAL-2 antagonizes this inhibitory effect of SYP proteins on PC function.

Second, we found evidence that significant homolog pairing occurred in *hal-2* mutants that lacked the SYP proteins. The *syp* mutants are proficient for establishment of pairing at PCs and can achieve a significant degree of lengthwise homolog alignment, but are defective in stabilization and maintenance of homologous associations [10], [28], [34]–[36]; this made it possible to test whether significant homolog pairing occurred in *hal-2; syp* double mutants. We quantified levels of pairing at the X chromosome PCs (using IF for HIM-8), at chromosome V PCs (V-PCs) (using IF for ZIM-2) and at an internal position of chromosome V (using FISH for the 5S rDNA locus) (Figures 4D–F). For all of these analyses, gonads were divided into 5 zones of equal lengths, as indicated in Figure 4C.

Pairing at the X-PC was quantified from Zones 2–5 (Figure 4D), as HIM-8 localization at the X-PCs is most robust from the TZ through late pachytene [13]. Whereas pairing at the X-PCs was effectively abolished in the *hal-2* single mutant, high levels of pairing were restored in the *hal-2; syp-2* double mutant, with X-PC pairing levels peaking in Zones 3 and 4 at levels approaching those of *syp-2* and wild-type controls (Figure 4D). Although X-PC pairing was substantially restored in the *hal-2; syp-2* double mutant, however, pairing in all zones was significantly lower than in the *syp-2* single mutant, suggesting that restoration of synapsis–independent maintenance of PC pairing may be incomplete.

ZIM-2 pairing levels were analyzed specifically for Zone 2, the zone where ZIM-2 foci are most prominently detected during normal meiosis (Figure 4E). In wild-type gonads, Zone 2 includes some premeiotic nuclei, the TZ, and some early pachytene nuclei; hence, the wild-type data set included nuclei with two bright ZIM-2 foci, nuclei with a single focus (indicative of paired PCs), and some nuclei without ZIM-2 foci. As described above, *hal-2* mutants lack nuclei with clear bright ZIM-2 foci, and these foci are restored in the *hal-2; syp-2* double mutants (Figure 4B). Among the nuclei with ZIM-2 foci in Zone 2, the proportion of nuclei in which foci were paired in the *hal-2; syp-2* double mutant was similar to the proportions observed for both wild-type and *syp-2* mutant gonads (Figure 4E), indicating highly successful pairing at the V-PCs in early meiotic prophase nuclei with ZIM-2 foci in the *hal-2; syp-2* double mutants. Whereas ZIM-2 foci (and other markers of PC-mediated movement) persisted into Zones 3 and 4 in *syp-2* mutant gonads, ZIM-2 foci were not detected beyond Zone 2 in the *hal-2; syp-2* double mutants (data not shown), thus precluding assessment of pairing at V-PCs throughout a larger portion of the *hal-2; syp-2* mutant germ lines.

Pairing at the 5S locus on chromosome V was assessed for all five zones of the gonad (Figure 4F). While pairing at this locus was abolished throughout the gonad in the *hal-2* single mutant, the *syp-3; hal-2* double mutant exhibited a highly significant increase in pairing over the *hal-2* single mutant specifically in Zone 2 (p<0.0001), the same zone in which chromosome V PCs were shown to be active in the *hal-2; syp-2* double mutant. However, increases in pairing were not detected in other zones and pairing levels for *syp-3; hal-2* for Zones 2–5 were consistently significantly lower than in *syp-3* controls. As markers of active PCs persist beyond Zone 2 at the X-PCs but not at the autosomal PCs (in both *hal-2; syp-2* and wild-type), it is likely that the lower level of pairing restoration observed for chromosome V upon removal of SYP proteins reflects a difference between the X chromosome and autosomes in the duration of PC activity.

Taken together, our analyses show that significant homologous pairing at the PCs can occur in *hal-2* mutants when SYP proteins are absent, indicating that the SYP proteins can interfere with pairing, likely through inappropriate association with unaligned chromosomes. These findings imply that HAL-2 promotes PC function by counteracting inhibitory effects of the SYP proteins. However, incomplete restoration of pairing also indicates that HAL-2 plays additional role(s) in homolog alignment beyond antagonizing inhibitory effects of SYP proteins on PC function (see below).

### *hal-2* mutants exhibit reduced chromosomal localization of HTP-1/2 that is rescued by removal of SYP proteins

**Figure 5:**
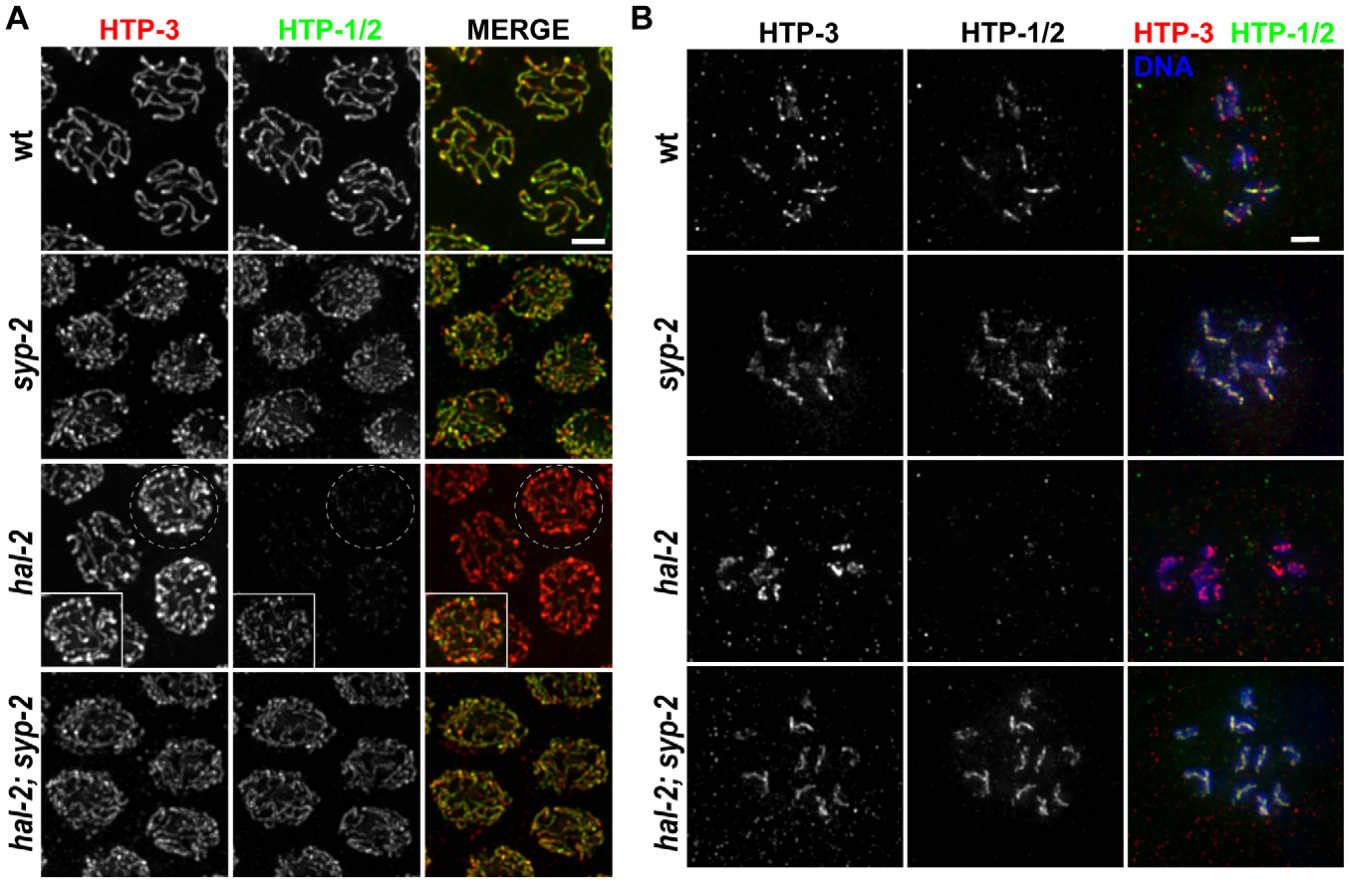
*hal-2* mutants exhibit reduced chromosomal localization of HTP-1/2 that is rescued by removal of SYP proteins. (A) Co-immunolocalization of LE components HTP-3 and HTP-1/2 in pachytene nuclei of indicated genotypes. HTP-1/2 and HTP-3 are detected colocalized along the full lengths of paired and synapsed homologs in wild-type nuclei and along the axes of unpaired chromosomes in nuclei from the late pachytene region in a *syp-2* single mutant; HTP-1/2 and HTP-3 IF signals are present in similar ratios in these two genotypes. Images from the *hal-2* mutant show that the level of HTP-1/2 relative to HTP-3 was greatly reduced compared to wild-type and *syp-2* controls; however, HTP-1/2 localization was not abolished in the *hal-2* mutant, as shown in the inset, in which the HTP-1/2 IF signal has been adjusted to highlight the faint HTP-1/2 staining along the axes of the unpaired chromosomes. HTP-1/2 localization in *hal-2; syp-2* double mutant nuclei appears similar to the *syp-2* single mutant. Bar, 2 µm. (B) Each panel depicts the chromosomes of a single diakinesis oocyte of the indicated genotype, co-stained for HTP-1/2 and HTP-3. HTP-1/2 is detected on the chromosomes in the wild-type and *syp-2* control oocytes, but is not detected on the chromosomes in the *hal-2* mutant oocyte; HTP-1/2 staining is detected on the chromosomes in the *hal-2; syp-2* oocyte, reflecting rescue of the HTP-1/2 localization defect in *hal-2* mutants by the removal of SYP proteins. Bar, 2 µm.

*hal-2* mutants show several phenotypic similarities with worms lacking both HTP-1 and HTP-2 (LE components that are paralogs of HIM-3 and HTP-3), including association of SYP proteins with the axes of unpaired chromosomes in pachytene nuclei [33], [37]. Thus, we assessed localization of HTP-1/2 in *hal-2* mutant gonads by IF. (Since HTP-1 and HTP-2 are recognized by the same antibody, they are referred to as HTP-1/2 [37].) In wild-type germ lines, HTP-1/2 colocalize with other SC components (including the SYP proteins) along the full lengths of paired homologs until late pachytene, when HTP-1/2 and SYP proteins begin to become redistributed in a crossover-dependent fashion to reciprocal chromosomal domains such that their localization is almost mutually exclusive by diakinesis, with HTP-1/2 localizing on the long arms of the bivalent and SYP proteins localizing on the short arms (Figure 5) [37], [38]. The localization of HTP-1/2 on diakinesis chromosomes is markedly different from the other axis components, such as HTP-3, which localize to all four arms of the bivalent (Figure 5B) [27], [37].

We found that HTP-1/2 localization was impaired in the *hal-*2 mutant. During the pachytene stage, faint HTP-1/2 staining was detected along the axes of the unpaired chromosomes, but the level of HTP-1/2 relative to HTP-3 was much lower than in wild-type controls (Figure 5A). Further, while HTP-3 was present on univalent chromosomes of diakinesis-stage oocytes in the *hal-2* mutant, HTP-1/2 staining was undetectable (Figure 5B).

The fact that SYP proteins load improperly onto unaligned chromosome axes in *hal-2* mutants raised the possibility that the SYP proteins might be preventing the normal localization of HTP-1/2. Consistent with this hypothesis, HTP-1/2 localization was substantially restored in *hal-2; syp-2* double mutant gonads, both at the pachytene and diakinesis stages (Figure 5). These results provide additional support for a previously proposed idea that SYP proteins and HTP-1/2 are incompatible, each antagonizing the chromosomal localization of the other [37]. Further, the data suggest that HAL-2 plays a role in promoting a temporary compatibility between SYP proteins and HTP-1/2 that enables them to coexist along the lengths of chromosomes from zygotene through mid-pachytene stages during wild-type meiosis.

### *hal-2* encodes a protein that concentrates in the nucleoplasm of germ cells

**Figure 6:**
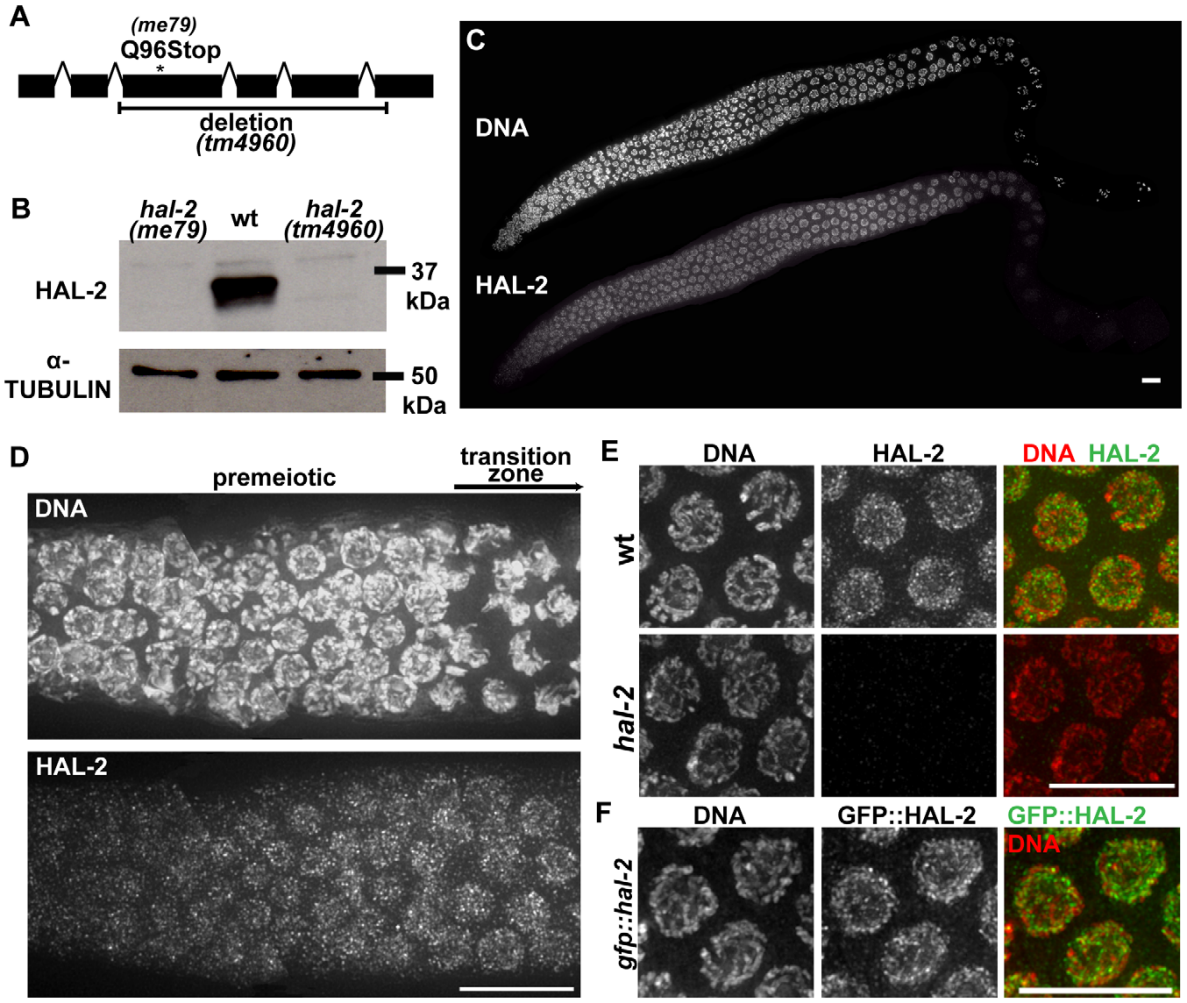
*hal-2* encodes a protein that concentrates in the nucleoplasm of germ cells. (A) Schematic diagram of *hal-2* gene structure depicting the locations of the nonsense mutation and the 761 bp deletion in the *me79* and *tm4960* alleles. (B) Western blot of whole worm lysates from the indicated genotypes probed with α-HAL-2 and α-α-TUBULIN (loading control) antibodies. A band corresponding to the expected 36.1 kDa HAL-2 protein is detected in wild-type lysates and is absent from both *me79* and *tm4960* mutant lysates. (C) Low magnification image of a wild-type whole mount germ line stained with α-HAL-2. HAL-2 staining is first observed in the premeiotic nuclei preceding the TZ, is present in nuclei throughout the TZ and pachytene regions, and progressively weakens during diplotene. Bar, 10 µm. (D) High magnification image of HAL-2 immunolocalization in a portion of a wild-type germ line extending from the premeiotic zone to the TZ; comparison of the DNA staining and HAL-2 staining within the TZ reflects the fact that HAL-2 is concentrated predominantly in the nucleoplasm. Bar, 10 µm. (E) IF images of HAL-2 in wild-type and *hal-2* pachytene nuclei. HAL-2 immunostaining does not colocalize with DAPI-stained chromatin and is distributed broadly within the nucleoplasm of wild-type nuclei. (Some short linear stretches are apparent within this nucleoplasmic HAL-2 staining, but they do not correspond to a known structure and their significance is unclear.) HAL-2 immunostaining is absent in *hal-2* mutant nuclei, demonstrating the specificity of the antibody and the lack of full-length HAL-2 protein in the mutant. Bar, 10 µm. (F) Immunolocalization of GFP::HAL-2 in pachytene nuclei of worms containing a functional GFP::HAL-2 transgene. GFP::HAL-2 localized to the nucleoplasm of germ cells, in agreement with the localization pattern obtained from α-HAL-2 IF. Bar, 10 µm.

Mapping of the *hal-2(me79)* mutation, phenocopy by RNAi, and identification of a nonsense mutation in the *me79* allele by sequence analysis identified *T16H12.11* as the *hal-2* gene (see Materials and Methods) (Figure 6A). An independently generated deletion allele, *tm4960*, is missing 596 bp of the 927-bp coding region (Figure 6A) and fails to complement *me79*, confirming the molecular identity of *hal-2* as *T16H12.11*. The two alleles cause similar phenotypic defects and both are likely null alleles; all of the analyses reported here were performed using the *me79* mutant unless otherwise specified. Although the *T16H12.11* gene model in WormBase (release WS227) predicts a 295 aa protein, we propose a modified gene model (that uses an upstream ATG) based on upstream conservation of amino acid sequence in other *Caenorhabditis* orthologs (Figure S8). The revised gene model encodes a predicted 308 aa protein with MW of 36.1 kDa. An antibody raised against the C-terminal 100 amino acids of HAL-2 detects a band of slightly less than 37 kDa in Western blot analysis of wild-type whole worm lysates, consistent with the protein size predicted by our revised gene model. This band was absent in lysates from both *hal-2(me79)* and *hal-2(tm4960)* mutant worms (Figure 6B), indicating that no full-length protein is produced in these mutants.

Bioinformatics analyses using PSI-BLAST and several protein structure prediction servers yielded little information regarding HAL-2 function. No homologs were detected outside of the *Caenorhabditis* genus, and no conserved motifs were detected by searches of the Conserved Domain Database. Further, while HAL-2 orthologs were found in other *Caenorhabditis* species, they exhibited relatively low sequence conservation (52% amino acid identity between ortholog pairs). This differs from conservation levels observed within *Caenorhabditis* for proteins implicated directly in meiotic recombination (which range from 74–98% amino acid identity) and is more in line with conservation levels for SYP proteins ortholog pairs (which range from 41–61% amino acid identity). Analysis using the COILS and Paircoil2 programs [39], [40] suggests that a short domain (33–46 amino acids) in HAL-2 orthologs may have the capacity to adopt a coiled-coil conformation, although the probability of this conformation differed widely among HAL-2 orthologs and reached significance threshold only for a subset of them.

IF analysis using our α-HAL-2 antibody demonstrated that the HAL-2 protein concentrates in the nuclei of germ cells. HAL-2 staining was first detected in germ cell nuclei before they entered the TZ and gradually weakened during diplotene until no signal was observed by the end of diakinesis (Figures 6C–E). As the majority of the IF signal did not colocalize with DAPI-stained chromatin, nucleoli (data not shown) or SC (see below), we infer that HAL-2 is predominantly nucleoplasmic. This nucleoplasmic staining was detected in wild-type gonads but was absent in *hal-2* mutant gonads (Figure 6E), further verifying the specificity of the antibody. We also generated a functional GFP::HAL-2 transgene that rescues the Him and progeny lethality phenotypes of *hal-2* mutants (Table S1). GFP::HAL-2 expressed from this transgene exhibited a similar nucleoplasmic localization in germ cells, although the protein persisted beyond diplotene (Figure 6F and data not shown), likely because of heterologous promoter and 3′UTR sequences.

### HAL-2 colocalizes with SYP proteins in aggregates that form when SC assembly is prevented

**Figure 7:**
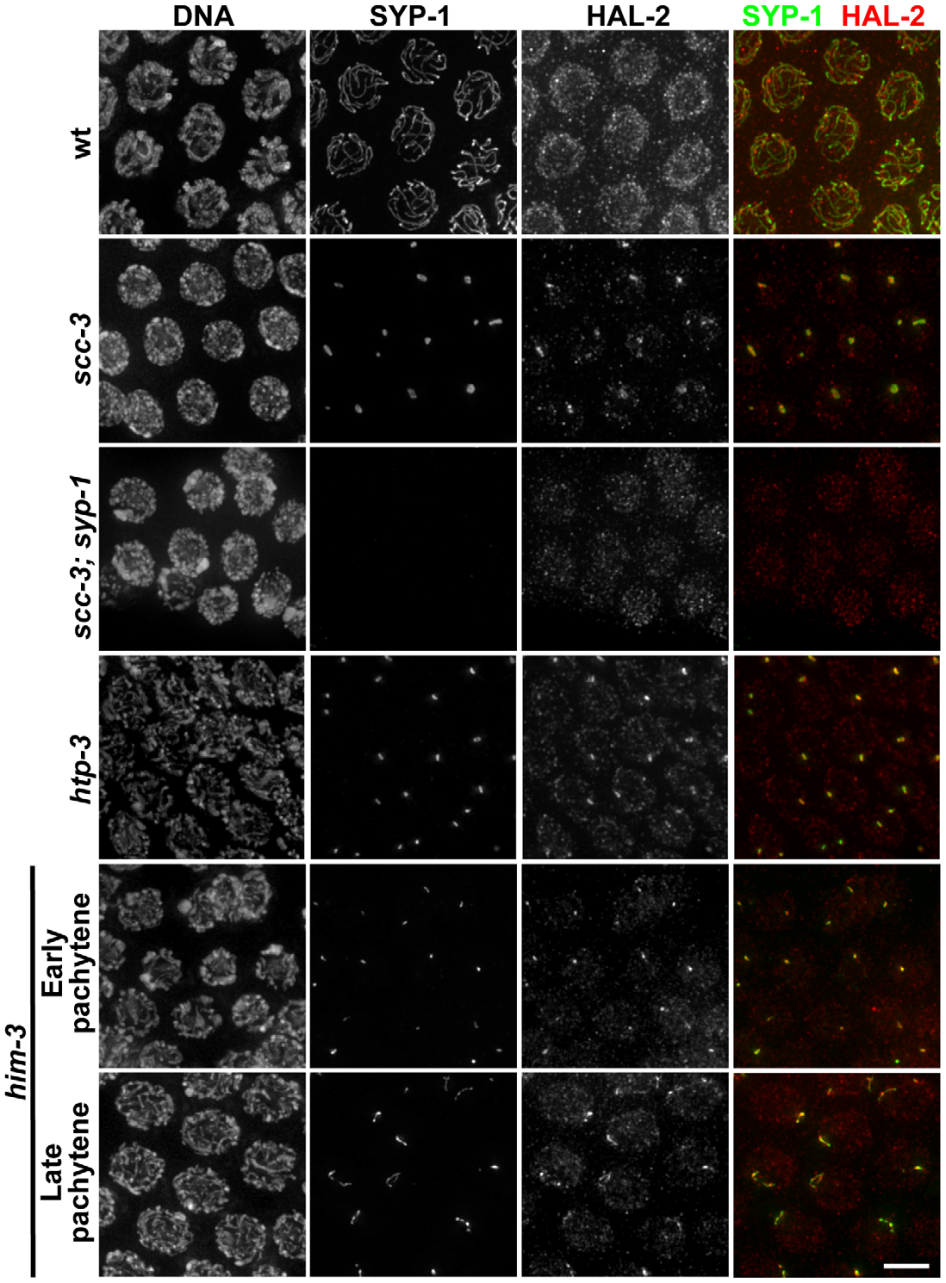
HAL-2 colocalizes with SYP proteins in aggregates that form when SC assembly is prevented. Co-staining of SYP-1 and HAL-2 in pachytene nuclei of indicated genotypes. In the wild-type nuclei, HAL-2 is localized to the nucleoplasm and exhibits very little colocalization with SYP-1, which is localized at the interface between paired homologs. In contrast, in mutants with severely disrupted SC assembly (cohesin mutant *scc-3(ku263)* and mutants lacking LE components HTP-3 or HIM-3), SYP-1 does not localize along chromosomes but instead becomes concentrated in nuclear aggregates (presumably polycomplexes); in these mutants, HAL-2 is detected in the nucleoplasm but also colocalizes with SYP-1 in the nuclear aggregates. Further, localization of HAL-2 in nuclear aggregates in *scc-3* mutants is SYP-dependent. Bar, 4 µm.

Since our phenotypic analyses indicated that HAL-2 plays an important role in restraining the behavior of the SYP proteins, we used IF to investigate their spatial relationship *in situ*. In wild-type germ cell nuclei, little or no overlap was detected between the localization of HAL-2, which concentrates in the nucleoplasm, and SYP-1, which concentrates at the interface between aligned chromosomes (Figure 7).

However, when we analyzed the localization of HAL-2 in the *scc-3* cohesin mutant, in which chromosome axes and SC assembly are severely disrupted and LE precursors (including the other cohesin subunits) and SYP proteins form aggregates (presumably polycomplexes) in the nuclei instead of loading extensively onto chromosomes, we detected HAL-2 both in the nucleoplasm and concentrated together with SYP-1 in the nuclear aggregates (Figure 7) [27], [41]. Localization of HAL-2 in nuclear aggregates is dependent on SYP-1, as HAL-2 nuclear aggregates are not observed in *scc-3; syp-1* germ cell nuclei (Figure 7). Further, we found that HAL-2 colocalized with SYP-1 in nuclear aggregates in an *htp-3* mutant, in which LEs do not assemble along the chromosomes but cohesin components are also not present in the nuclear aggregates (Figure 7) [42]. Finally, we examined HAL-2 localization in a *him-3* null mutant, which does assemble LEs that contain cohesin complexes, HTP-1/2 and HTP-3, but is severely defective for SC assembly [26], [27], [37], [42]. In the *him-3* mutant, SYP-1 appears as small nuclear aggregates at early pachytene that elongate into short stretches by late pachytene, and HAL-2 was found both in the nucleoplasm and colocalized together with SYP-1 in these structures (Figure 7). HAL-2 is not required for formation of these SYP-1 nuclear aggregates, however, as SYP-1 aggregates were still formed in *hal-2; him-3* double mutants (Figure S9). Together, these analyses of SYP-dependent localization of HAL-2 to nuclear aggregates in various mutants with defective LE and SC assembly raise the possibility that HAL-2 might interact with SYP proteins or other SC-associated proteins; however these IF analyses do not address whether such potential interactions are direct or indirect.

### HAL-2 has additional roles in meiosis beyond preventing inappropriate association of SYP proteins with chromosomes

**Figure 8:**
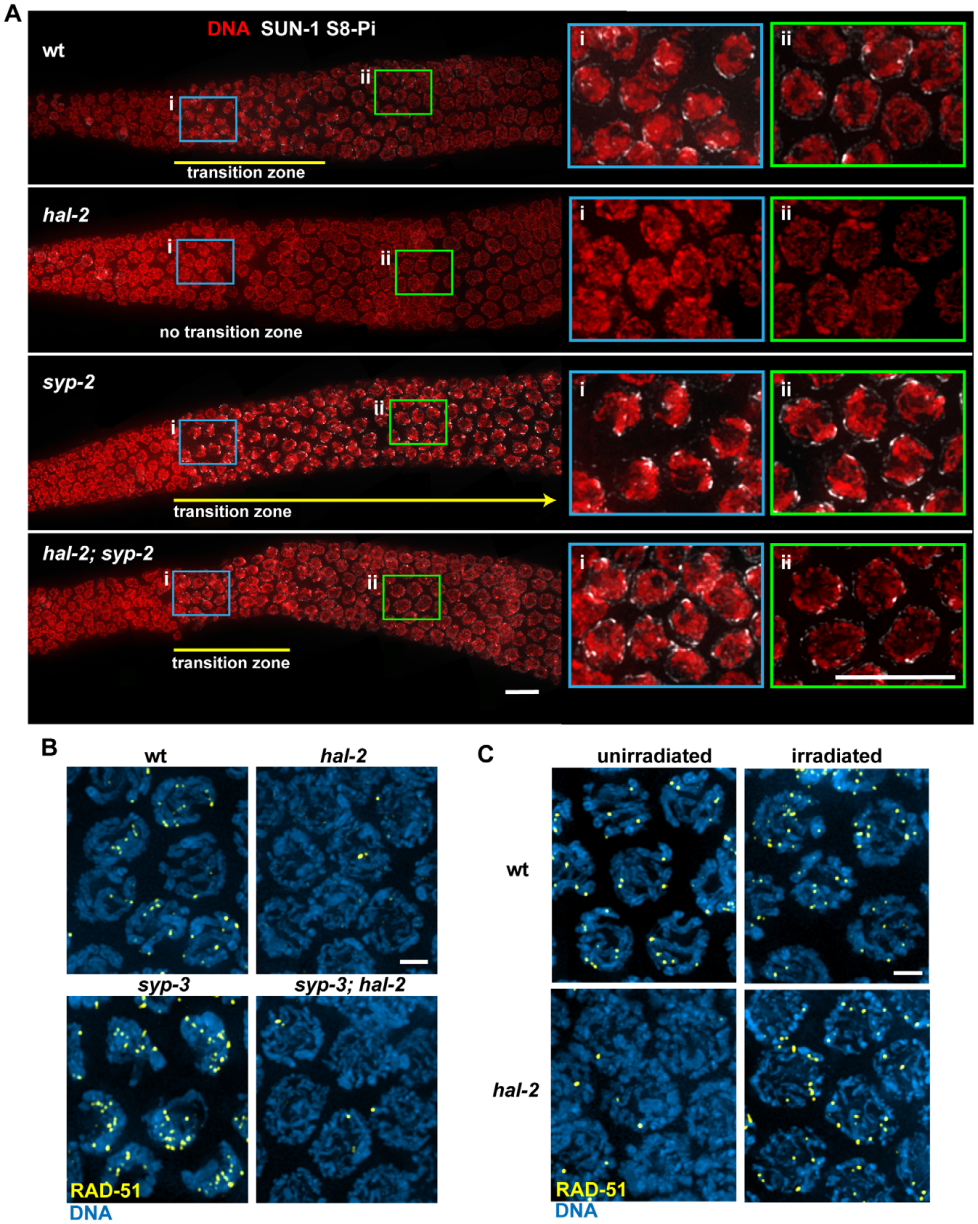
HAL-2 has additional roles in meiosis beyond preventing inappropriate association of SYP proteins with chromosomes. (A) Immunolocalization of SUN-1 S8-Pi in DAPI-stained gonads of the indicated genotypes, with meiosis progressing from left to right. Panels on the right are zoomed-in images of early (i; blue box) and later (ii; green box) stages of meiotic prophase. In the wild-type gonad, nuclei in the TZ (i) exhibit a clustered chromosome configuration and have multiple SUN-1 S8-Pi NE patches, whereas after progression into pachytene (ii), the chromosomes exhibit a more dispersed distribution within the nuclei and most nuclei have only a single SUN-1 S8-Pi focus remaining on the NE. In the *hal-2* mutant gonad, nuclei from both regions (i and ii) have dispersed chromosomes and either lack SUN-1 S8-Pi (i) or have low levels of diffuse SUN-1 S8-Pi NE signal (ii; Figure S10), while in the *syp-2* mutant, nuclei from both regions exhibit clustered chromosomes and multiple SUN-1 S8-Pi NE patches, reflecting prolonged persistence of chromosome mobilization. The *hal-2; syp-2* double mutant gonad does not exhibit persistent chromosome clustering and multiple persistent SUN-1 S8-Pi NE patches; chromosome clustering is restricted to a short TZ (i), and only a single SUN-1 S8-Pi NE focus persists beyond the TZ, in nuclei with dispersed chromosomes (ii), similar to wild-type gonads. Yellow lines depict the extent of the TZs, with the yellow arrow indicating extension of the TZ in the *syp-2* mutant beyond the region shown in the image. The *zyg-12::gfp* transgene was present in the gonads shown here. Bars, 10 µm. (B) Immunostaining of DNA strand exchange protein RAD-51 in early/mid-pachytene nuclei. While abundant RAD-51 foci are observed in both wild-type nuclei and *syp-3* mutant nuclei, very few foci were detected in the *hal-2* and *syp-3; hal-2* mutants, with most nuclei lacking RAD-51 foci. Bar, 2 µm. (C) Immunostaining of RAD-51 in mid-pachytene nuclei of *hal-2* mutants and wild-type controls. Abundant RAD-51 foci are observed in nuclei of *hal-2* germ lines exposed to 1 krad of γ irradiation, while few foci are detected in the nuclei of unirradiated *hal-2* controls. Bar, 2 µm.

Consistent with our finding that homolog pairing in the *hal-2; syp* double mutants is not restored to the levels observed in *syp* single mutants, closer examination of DAPI-stained chromosome morphology, SUN-1 phosphorylation, ZYG-12::GFP patches and PLK-2 localization in *hal-2; syp-2* gonads provided further evidence that HAL-2 has additional roles in meiosis beyond regulating SYP loading (Figures 8A, S10 and data not shown). In contrast to the dramatically extended TZ observed in *syp-2* mutant gonads, in which nuclei with clustered chromosomes and multiple bright NE patches reflecting ongoing chromosome mobilization persist until near the very end of the pachytene region (Figure 8A) [14], [15], [21], [34], *hal-2; syp-2* mutant gonads lack persistent chromosome clustering, and only the single NE focus associated with HIM-8 persists beyond the TZ, as in wild-type gonads (Figures 8A and S7). The fact that *hal-2; syp-2* nuclei can exit from the clustered chromosome organization despite lack of synapsis suggests that HAL-2 may be required for normal functioning of a checkpoint-like mechanism that operates to make redispersal of clustered chromosomes contingent upon SC assembly [18].

We also found that *hal-2* mutants exhibit severely reduced levels of immunostaining for DNA strand exchange protein RAD-51 [34], [43]. Whereas multiple RAD-51 foci were detected in most early/mid-pachytene nuclei in wild-type germ lines, most nuclei in *hal-2* mutant germ lines lacked RAD-51 foci (Figure 8B), suggesting that initiation of recombination may be impaired. Further, this deficit of RAD-51 foci was not suppressed in the *syp-3; hal-2* double mutant, which also lacked RAD-51 foci in most nuclei, indicating that this phenotype is not a secondary consequence of inappropriate SYP loading. The reduced levels of RAD-51 foci in the *syp-3; hal-2* double mutant contrasts with the increased levels of foci observed in the *syp-3* single mutant (Figure 8B) [44], indicating that absence of HAL-2 prevents accumulation of RAD-51 foci. Reduction in RAD-51 foci in *hal-2* mutants suggests either that *hal-2* mutants have a reduced number of double-strand DNA breaks (DSBs) or that HAL-2 is needed for efficient loading of RAD-51 onto DSBs. To test whether HAL-2 is required for accumulation of RAD-51 on DSBs, we induced DSBs with ionizing irradiation in *hal-2* animals and dissected them 1 hour post irradiation for RAD-51 immunostaining. Abundant RAD-51 foci were detected in nuclei throughout the irradiated *hal-2* mutant germ lines (Figure 8C), indicating that HAL-2 is not required for accumulation of RAD-51 on DSBs. Taken together, these data suggest that HAL-2 has a role in promoting initiation of recombination that is distinct from its role in regulating SYP loading.

Variable defects in PC-mediated chromosome clustering in combination with impaired DSB formation had been reported previously for the *C. elegans him-19* [45] mutant. Moreover, some of the defects in PC function in *him-19* mutants were rescued by the introduction of DSBs by irradiation [45], raising the possibility that defects in PC function in *hal-2* mutants might be similarly rescued by artificial induction of DSBs. We tested this by inducing DSBs in *hal-2* mutants by ionizing irradiation and dissecting the animals 2 hours post irradiation for IF analysis. No chromosome clustering, recruitment of bright ZIM-3 foci to the PCs and nuclear envelope, or NE patches of phosphorylated SUN-1 S12-Pi were observed in the irradiated *hal-2* gonads (Figure S11), indicating that these defects in the *hal-2* mutant are not secondary consequences of its defect in recombination initiation.

## Discussion

### Inhibitory potential of SYP proteins on homologous pairing

The work reported here highlights the potential of SC central region (CR) precursors to exert detrimental effects that interfere with the process of homolog pairing. This potential emphasizes the need for SC precursors to be carefully shepherded during early meiotic prophase to avoid antagonizing the very process that they normally serve to stabilize. One way that CR precursors can cause trouble is by inappropriately linking the axes of nonhomologous chromosomes. If transient nonhomologous interactions that occur during early prophase are stabilized by synapsis, chromosomes will then have fewer chances to associate and pair with their correct partners. This is illustrated by the improved pairing observed when SYP proteins (and nonhomologous synapsis) were eliminated in a mutant with impaired SUN-1 function [16]. Nonhomologous synapsis may also prevent and/or restrain the active prophase chromosomal movements that promote pairing, as illustrated by the amelioration of restrained SUN-1::GFP NE patch movement in an *htp-1* mutant by the removal of SYP proteins [21]. Our comparative analyses of *hal-2; syp* double mutants with *hal-2* single mutants have revealed several additional ways in which CR precursors can be detrimental.

Firstly, SYP proteins can interfere with pairing by inhibiting CHK-2-dependent PC activities at the onset of meiotic prophase. In *hal-2* mutants, NE protein SUN-1 is not phosphorylated on Ser12 (and S8-Pi is severely reduced and/or delayed), PCs fail to recruit PLK-2, autosomal PC-binding proteins fail to concentrate at their respective PCs or the NE, and no ZYG-12::GFP NE patches or PC-mediated chromosome movements are observed. Coordinate loss of these features suggests that lack of pairing in *hal-2* mutants likely results primarily from the abrogation of vital CHK-2-dependent PC functions. Moreover, the fact that all of these problems are alleviated by removal of SYP proteins implies that the unconstrained behavior of SYP proteins in the absence of HAL-2 is responsible for preventing PC function in this context.

Secondly, SYP proteins can interfere with proper chromosomal association of HORMA domain proteins HTP-1/2. An incompatibility between CR proteins and (some) meiosis-specific HORMA domain proteins is evident from observations of apparently opposing spatial relationships of these classes of proteins along chromosomes in several different organisms. In *S. cerevisiae*, HORMA protein Hop1 and CR protein Zip1 exhibit largely complementary patterns of high and low abundance along pachytene chromosomes [46]. Further, mouse HORMAD1 and HORMAD2 lose their chromosomal localization upon installation of CR protein SYCP1 [47]. In *C. elegans*, all four HORMA proteins (HTP-3, HIM-3 and HTP-1/2) coexist with the SYP proteins along the lengths of chromosomes for most of the pachytene stage, but at late pachytene HTP-1/2 and SYP proteins are triggered by (nascent) COs to relocalize to reciprocal chromosomal domains [27], [37], [38]. These patterns all suggest a mutually antagonistic relationship between CR proteins and (some) HORMA proteins. In *C. elegans*, the coexistence of HTP-1/2 and SYP proteins from zygotene until late pachytene further implies a mechanism that temporarily counteracts this antagonism. The temporary compatibility between these proteins during early prophase requires HAL-2, as our data show that the unconstrained behavior of SYP proteins in *hal-2* mutants inhibits axis association of HTP-1/2 during early prophase. Since HTP-1/2 is essential for homolog pairing [18], [33], reduced chromosomal HTP-1/2 likely contributes to the pairing defect in *hal-2* mutants.

### Regulatory role(s) of HAL-2 in controlling behavior of CR precursors in early prophase

Taken together, our data indicate that HAL-2 plays a major role in homolog pairing by restricting the detrimental behavior of SYP proteins and constraining their loading to the appropriate context. Moreover, the contributions of HAL-2 to the coordination of pairing and synapsis are distinct from and parallel to the contribution of a previously identified checkpoint-like mechanism that ensures that SC central region assembly is contingent upon successful homology verification [14], [18]. LE component HTP-1, NE protein SUN-1, and dynein-mediated chromosome movements were shown to be involved in this mechanism [14], [16], [18]. Inhibition of dynein causes a significant delay between pairing and SYP loading (implying that motion and/or exertion of tension is required to license SYP loading), while HTP-1 loss or impaired SUN-1 function bypasses the dynein requirement and leads to nonhomologous synapsis (suggesting that these proteins are required to generate or respond to a “wait synapsis” signal). In contrast, neither pairing nor nonhomologous synapsis occurs in *hal-2* mutants, and instead, SYP proteins load along the axes of individual unpaired chromosomes. This suggests the possibility that HAL-2 might function in promoting maturation of CR precursors, such that upon licensing of SC assembly, SYP installation invariably occurs in a manner that links LEs together. Thus, HAL-2 may have two potentially inter-related roles in regulating the behavior of SYP proteins: i) shepherding/restraining CR subunits to prevent them from wreaking havoc in the nucleus and ii) enabling their timely maturation into a form that can be assembled cooperatively into mature SCs.

These proposed roles suggest that HAL-2 might serve in the capacity of a chaperone to help ensure timely and appropriate SC assembly. Although the HAL-2 sequence does not identify it as a member of previously known chaperone families, there are numerous striking parallels between HAL-2 and Fpr3, a prolyl isomerase that regulates SC assembly during budding yeast meiosis [48]. Like HAL-2, Fpr3 localizes in the nucleoplasm of meiotic cells and colocalizes with CR precursors in polycomplexes that form when SC assembly is prevented [48]. Further, like HAL-2, Fpr3 functions in parallel with other mechanisms to prevent assembly of CR precursors onto chromosomes until licensing conditions have been met [48]. Thus, both HAL-2 and Fpr3 can be seen as guardians of CR precursors, inhibiting their inappropriate behavior.

The cooperative and processive nature of SC assembly and the inherent tendency for self-assembly of its precursors [1] present a challenge for meiotic cells: they must accumulate large pools of precursors needed to accomplish rapid SC assembly, while at the same time preventing these precursors from aggregating into nonfunctional structures and/or interfering with prerequisite events. In *C. elegans*, the SC central region consists of four interdependent coiled-coil SYP proteins that interact to span the distance between the LEs [10], [28], [29], [34], [35]. Thus, involvement of regulatory proteins not included in the mature SC (such as HAL-2) represents a practical solution for controlling maturation of these CR precursors and constraining their loading to occur only in the appropriate context.

In addition to HAL-2, assembly of the SC central region also requires CRA-1 [49], the *C. elegans* ortholog of the non-catalytic subunit of the NatB N-terminal acetyltransferase complex, which acetylates the N-terminal methionine of proteins containing Met-Asp-, Met-Glu- or Met-Asn- [50], [51]. As in *hal-2* mutants, SYP proteins associate with unpaired LEs in *cra-1* mutants [49], raising the possibility that maturation of SC precursors may require N-terminal acetylation of one or more subunits. While we have also suggested a role for HAL-2 in promoting CR subunits maturation, it is clear that HAL-2 and CRA-1 make different contributions to regulating SYP behavior. Loading of SYP proteins onto chromosome axes in *cra-1* mutants occurs later than in *hal-2* mutants and does not interfere with homolog pairing [49]. Further, whereas association of SYP proteins with unpaired chromosomes in *cra-1* mutants is dependent on DSB formation and progression of meiotic DSB repair processes [49], SYP proteins load onto unpaired chromosomes in *hal-2* mutants in the absence of DSBs (as in *hal-2; spo-11* double mutants; Figure S5), indicating that the chromosomal association of SYP proteins in *hal-2* mutants is not dependent on DSBs or recombination intermediates.

### Potential role of HAL-2 in coupling chromosome redispersal with SC assembly

In addition to the checkpoint-like mechanism that couples SC assembly to homology verification, a related but distinct mechanism operates during *C. elegans* meiosis to render redispersal of clustered chromosomes and termination of active chromosome motion contingent upon SC assembly [18], [28]. HAL-2 may also play a role in this coupling mechanism that is distinct from its role in preventing SYP proteins from interfering with pairing. This is suggested by the fact that chromosome clustering and PC-mediated chromosome mobilization do not persist in *hal-2; syp* double mutants, in which synapsis cannot occur. It has been postulated that chromosome redispersal is regulated by a signaling mechanism that monitors synapsis progression, and HTP-1 has been suggested to be involved in generating and/or responding to an inhibitory “wait dispersal” signal that maintains chromosome clustering [18]. Since HTP-1/2 loading onto chromosomes is largely restored by removal of SYP proteins (in *hal-2; syp* double mutants), the inability to maintain chromosome clustering is not a consequence of lack of HTP-1 in the *hal-2* mutant, but it may reflect a cooperation of HAL-2 with HTP-1 in regulating or responding to the “wait dispersal” inhibitory signal.

Implicit in such a coupling mechanism is that progression of synapsis must generate “start dispersal” activation signals to counteract the existing inhibitory signals. The SYP proteins themselves likely contribute, as suggested by analysis of the *syp-3(me42)* mutant, which expresses C-terminally truncated SYP-3 [28]. In contrast to the persistent chromosome clustering observed in *syp* null mutants, a premature exit from chromosome clustering is observed in the *syp-3(me42)* mutant; it was proposed that association of SYP proteins with unpaired chromosomes in this mutant may act as the trigger for chromosome redispersal, as removal of other SYP proteins in the *syp-3(me42)* mutant restored persistent chromosome clustering [28]. This contrasts with the *hal-2* mutant, in which elimination of SYP proteins did not lead to persistent clustering, further supporting the notion that HAL-2 may play a SYP-independent role in the coupling mechanism and/or the maintenance of chromosome movement.

## Materials and Methods

### Genetics

*C. elegans* strains were cultivated at 20°C under standard conditions [52], unless otherwise specified. For experiments involving meiotic mutants, homozygous mutants were picked from the progeny of heterozygous balanced parents by the absence of dominant markers associated with the balancers and/or by the presence of a recessive marker *cis*-linked with the mutant allele. For Figure S5, the *spo-11* single mutant controls imaged were heterozygous for the *hal-*2 mutation as they were siblings of *hal-2; spo-11* double mutants, both of which were derived from doubly balanced heterozygous parents. All experiments involving transgenic strains were performed in the presence of the wild-type gene unless otherwise noted. A list of strains used in this study is provided in Text S1.

*hal-2(me79)* was generated by EMS mutagenesis and isolated by screening for “Green eggs and Him” [19] and defects in prophase chromosome morphology and organization [20]. *hal-2(me79)* was mapped to a ∼126 kb region between positions 10057573 and 10183653 on chromosome III using standard crosses and SNP mapping [53]. The interval contained 28 known or predicted genes; complementation tests and RNAi led to the identification of *T16H12.11* as the likely *hal-2* gene. RNAi targeted to the conserved region of *T16H12.11* produced a partial *hal-2* phenocopy: no apparent TZ, unaligned chromosomes at pachytene and 8–10 DAPI-stained bodies at diakinesis. Sequencing of *T16H12.11* in the *hal-2(me79)* mutant revealed a C-to-T transition resulting in a premature stop (instead of glutamine) encoded at codon 96 of the 308 amino acid coding sequence.

*hal-2(tm4960)* was provided by Dr. S. Mitani at the *C. elegans* National BioResource Project, NIG, Japan; the 761 bp deletion was confirmed by PCR and sequencing.

### Cytological analysis

#### Immunofluorescence

Except for Figures 3C, S4, Video S3 and S4, immunostaining, DAPI-staining, image acquisition and processing using the DeltaVision deconvolution microscopy system were performed as in [18], except that slides were washed thrice in phosphate-buffered saline containing 0.1% Tween-20 (PBST) before blocking in 0.7% BSA. Dissections were performed on 20–24 hours post-L4 adults. A list of antibodies used is provided in Text S1. For experiments involving transgenic strains carrying GFP::SYP-3, GFP::HAL-2 or ZYG-12::GFP, L4 worms were placed at 25°C for 20–24 hrs before dissection.

For Figures 3C, S4, Video S3 and S4, gonads were dissected at 18 hours post L4 and fixed and processed for immunofluorescence as in [8]. Samples were stained with DAPI and mounted in glycerol containing 1% *n*-propyl gallate. Glass coverslips measured to 170±5 µm were placed over the samples and sealed with nail polish. 3D-SIM [30] was performed using a DeltaVision OMX microscope (Applied Precision), using an Olympus 1.4NA 100× UPlanSApo objective, with immersion oil (Cargille LaserLiquid) of refractive index 1.513, at 23–24°C. Image stacks of 8 µm thickness, 0.125 µm step size were acquired and processed to reconstruct high-resolution information. The videos each show a rotating series of 3D projections from a 3D-SIM data stack and were made with the IVE software package [54].

#### FISH

5S rDNA probes were amplified as in [36], [55] and labeled with Alexa Fluor 594 using the ULYSIS DNA labeling kit (Invitrogen). FISH was performed similarly to that in [20] with modifications described in Text S1.

Details of image acquisition and quantitative analysis of pairing are provided in Text S1.

### Live imaging

L4 animals were selected and placed at 25°C for 20 hours before dissection. Worms were dissected in 10 µl of M9 containing tricaine (0.1%), tetramisole (0.01%) and Hoechst 33342 (Invitrogen; 1∶1000) on a coverslip. The coverslip with the dissected worms was flipped onto a 2% agarose pad and imaged immediately using the DeltaVision deconvolution microscopy system with a 60× oil objective with 1.5× optivar. Images were acquired as stacks of 3 optical sections at 1.95 µm intervals every 15 s over a 5 min time period. Exposure times were kept constant for all images. Videos show maximum intensity projections of the 3D stacks displayed at 2 frames/s and were made with the Volocity (PerkinElmer) software.

### Irradiation experiments

For RAD-51 immunostaining, 20 hours post-L4 adults were exposed to 1 krad of γ irradiation from a Cs-137 source and dissected and fixed one hour post irradiation (21 hours post-L4). For ZIM-3 and SUN-1 S12-Pi immunostaining, 20 hours post-L4 adults were exposed to 5 krad of γ irradiation and dissected two hours post irradiation (22 hours post-L4). Unirradiated age-matched adults were dissected for immunostaining as controls for all irradiation experiments.

## Supporting Information

Figure S1 **SUN-1 S8-Pi is severely reduced and/or delayed in** ***hal-2*** **mutants.** Immunolocalization of SUN-1 S8-Pi in DAPI-stained gonads of the indicated genotypes, with meiosis progressing from left to right. In the meiotic prophase region of the wild-type germ line, SUN-1 S8-Pi immunostaining is most robust from the TZ to mid-pachytene stage and concentrates as bright NE patches and also localizes weakly throughout the NE (as shown in zoomed-in image). In contrast, no SUN-1 S8-Pi is detected in the corresponding region of the *chk-2* mutant germ line. Two representative examples of *hal-2* mutant germ lines are shown; SUN-1 S8-Pi is not detected in the regions corresponding to the transition zone of wild-type germ lines, and exhibits only diffuse NE localization (as shown in zoomed-in images) in a subset of nuclei in the early and mid-pachytene regions. Bar, 50 µm. Bar in zoomed-in images, 10 µm.
(PDF)

Figure S2 **HIM-8 localizes close to the nuclear envelope in a** ***hal-2*** **mutant.** IF images of hal-2 early prophase nuclei co-stained with antibodies against HIM-8 and nuclear lamin LMN-1, which localizes immediately adjacent to the inner surface of the NE. Images shown are 3D projections of partial data stacks in which sections from the tops and bottoms of the nuclei were omitted in order to illustrate the close proximity of the HIM-8 foci to the NE; not all HIM-8 foci were contained within the projections shown. Bar, 2 µm.
(PDF)

Figure S3 **Bright ZIM-3 foci and NE patches of ZYG-12::GFP and SUN-1 S8-Pi are restored in** ***hal-2; syp-2*** **double mutants.** (A) IF images of early prophase nuclei stained with antibodies against ZIM-3, the PC-binding protein specific for chromosomes I and IV. Bright ZIM-3 foci are detected near the NE of wild-type TZ nuclei (top panel), whereas the *hal-2* mutant nuclei lack clear ZIM-3 foci (middle panel). ZIM-3 foci are present in TZ nuclei of the *hal-2; syp-2* double mutant (bottom panel), indicating that the concentration of autosomal PC-binding proteins to bright NE-associated foci was restored. Bar, 5 µm. Animals used for these IF images contained the *zyg-12::gfp* transgene. (B) IF images of early prophase nuclei stained for ZYG-12::GFP and SUN-1 S8-Pi in *hal-2* and *hal-2; syp-2* germ lines. Chromosome clustering, SUN-1 Ser8 phosphorylation and NE patches containing ZYG-12::GFP and SUN-1 S8-Pi (features that are missing in the *hal-2* nuclei) are restored in the *hal-2; syp-2* nuclei. Bar, 2 µm. Animals used for these IF images contained the *zyg-12::gfp* transgene.
(PDF)

Figure S4 **Regions of fold-back near chromosome ends observed in** ***hal-2*** **mutants.** 3D-SIM images of a *hal-2* mutant pachytene nucleus stained for the LE component HTP-3 and CR protein SYP-1. A short stretch of parallel LEs flanking a single track of SYP-1 (as shown by right panel of zoomed-in images of region in white box) is detected in the *hal-2* mutant nucleus and corresponds to a region of fold-back near the end of the chromosome. Images shown are 3D projections of partial data stacks in which the bottom half of the nucleus was excluded in order to highlight the region of fold-back. Bars, 1 µm.
(PDF)

Figure S5 **SYP-1 localization in** ***hal-2; chk-2*** **,** ***hal-2; htp-1*** **and** ***hal-2; spo-11*** **double mutants resembles that in** ***hal-2*** **mutants.** IF images of HTP-3 and SYP-1 in late pachytene nuclei of the indicated genotypes. Images show: 1) many asynapsed chromosomes (HTP-3 stretches lacking SYP-1) and limited SYP-1 localized at the interface between nonhomologous chromosome segments in the *chk-2* mutant; 2) extensive synapsis (between paired X chromosomes and between nonhomologous chromosomes) in the *htp-1* mutant; 3) full homologous synapsis in the *spo-11* mutant; 4) SYP-1 localization in *hal-2; chk-2*, *hal-2; htp-1* and *hal-2; spo-11* double mutants, which appears similar to that observed in the *hal-2* single mutant, in that extensive SYP-1 is observed to be colocalized with HTP-3 along the axes of unpaired homologs. Bar, 5 µm.
(PDF)

Figure S6 **Markers of PC-mediated chromosomal movement in** ***syp-3; hal-2*** **mutants are dependent on** ***chk-2*** **.** IF images of early prophase nuclei stained with antibodies against SUN-1 S8-Pi reveal the presence of TZ nuclei with clustered chromosomes and SUN-1 S8-Pi NE patches in a *syp-3; hal-2* mutant (left panel) and the lack of these features in *syp-3; hal-2; chk-2* triple mutant nuclei (right panel), indicating that these markers of PC-mediated chromosomal movement are dependent on *chk-2*. Bar, 5 µm.
(PDF)

Figure S7 **A single ZYG-12::GFP focus associated with HIM-8 persists into pachytene of** ***hal-2; syp-2*** **mutants.** IF images of wild-type and *hal-2; syp-2* mutant nuclei of the indicated meiotic stages, co-stained for ZYG-12::GFP and HIM-8. In wild-type germ lines, a single ZYG-12::GFP NE focus associated with HIM-8 persists in nuclei that have exited the clustered chromosome configuration (right panels). Likewise, *hal-2; syp-2* double mutants exhibit TZ nuclei with multiple ZYG-12::GFP patches (circled nuclei in left panels), and after release from the clustered chromosome configuration, a single ZYG-12::GFP NE focus colocalizing with HIM-8 persists in pachytene nuclei (left and middle panels). Bar, 5 µm. Animals used for these IF images contained the *zyg-12::gfp* transgene.
(PDF)

Figure S8 **Modified gene prediction for** ***hal-2/T16H12.11***. (A) Modified predicted coding sequence for the *hal-2/T16H12.11* gene is shown in capital letters, with the additional coding sequence highlighted in yellow; upstream untranslated sequence is shown in lower case. (B) Alignment of the N-terminal portions of HAL-2 orthologs from five different *Caenorhabditis* species, showing strong conservation of the first N-terminal 13 amino acids. Red indicates identity among all five orthologs; blue indicates conservative substitutions; green indicates semi-conservative substitutions.
(PDF)

Figure S9 **SYP-1 forms aggregates and does not localize on chromosomes in** ***hal-2; him-3*** **double mutants.** IF images of pachytene nuclei from a *hal-2; him-3* double mutant, showing that SYP-1 is not loaded on chromosomes but does form nuclear aggregates, indicating that HAL-2 is not required for aggregate formation. Bar, 4 µm.
(PDF)

Figure S10 **Diffuse SUN-1 S8-Pi NE localization in** ***hal-2*** **mutant gonad shown in** **Figure 8A** **.** Immunolocalization of SUN-1 S8-Pi in gonads of the indicated genotypes, with meiosis progressing from left to right. Images shown are the same gonads as those shown in Figure 8A. In the *hal-2* mutant gonad shown, early meiotic prophase nuclei lack SUN-1 S8-Pi, and only a small subset of nuclei at a later stage of meiotic prophase exhibit diffuse SUN-1 S8-Pi NE signal, as demonstrated in the zoomed-in image (blue box). The SUN-1 S8-Pi signal was boosted in the zoomed-in image to illustrate the weak diffuse NE localization. Bars, 10 µm.
(PDF)

Figure S11 **Irradiation-induced DSBs do not restore chromosome clustering, bright NE-associated ZIM-3 foci or NE patches of SUN-1 S12-Pi in** ***hal-2*** **mutant gonads.** IF images of wild-type and *hal-2* mutant early prophase nuclei stained for SUN-1 S12-Pi and ZIM-3. As in unirradiated *hal-2* controls, chromosome clustering, ZIM-3 recruitment to PCs and the nuclear envelope, and formation of SUN-1 S12-Pi NE patches are not observed in *hal-2* gonads exposed to 5 krad of γ irradiation. Bar, 2 µm.
(PDF)

Table S1 **High embryonic lethality and Him phenotype in** ***hal-2*** **mutants are rescued by GFP::HAL-2.**
(DOC)

Text S1 **Supplemental materials and methods.**
(PDF)

Video S1 **ZYG-12::GFP patches move rapidly along the NE in wild-type TZ nuclei.** Live imaging of ZYG-12::GFP in Hoechst 33342-stained dissected wild-type worms, showing chromosome-associated ZYG-12::GFP patches moving rapidly along the NE of TZ nuclei. Videos are 3D projections of images captured at 15 sec intervals, displayed at 2 frames/sec. ZYG::GFP is in pink and DNA is in blue. Bar, 4 µm.
(MOV)

Video S2 **ZYG-12::GFP does not reorganize into moving NE patches in** ***hal-2*** **nuclei.** Live imaging of ZYG-12::GFP in Hoechst 33342-stained dissected *hal-2* worms reveals ZYG-12::GFP localization to the NE. However ZYG-12::GFP did not concentrate into the movement-competent NE patches observed in wild-type TZ nuclei. Videos are 3D projections of images captured at 15 sec intervals, displayed at 2 frames/sec. ZYG::GFP is in pink and DNA is in blue. Bar, 4 µm.
(MOV)

Video S3 **High-resolution 3D-SIM image of a wild-type pachytene nucleus.** A rotating series of 3D projections of the wild-type pachytene nucleus shown in Figure 3C, highlighting the two spatially resolved LEs of the paired homologs. HTP-3 is in green and HIM-8 is in red.
(MOV)

Video S4 **High-resolution 3D-SIM image of a** ***hal-2*** **mutant pachytene nucleus.** A rotating series of 3D projections of the *hal-2* mutant pachytene nucleus shown in Figure 3C, which exhibits single LEs rather than resolvable pairs of LEs as were observed in wild-type nuclei. HTP-3 is in green and HIM-8 is in red.
(MOV)

Video S5 **Movement-competent ZYG-12::GFP NE patches are restored in** ***hal-2; syp-2*** **nuclei.** Live imaging of ZYG-12::GFP in Hoechst 33342-stained dissected *hal-2; syp-2* worms showing the restoration of movement-competent ZYG-12::GFP NE patches in *hal-2; syp-2* TZ nuclei. Videos are 3D projections of images captured at 15 sec intervals, displayed at 2 frames/sec. ZYG::GFP is in pink and DNA is in blue. Bar, 4 µm.
(MOV)
