# Supplementary figures and images for: HAL-2 Promotes Homologous Pairing during Caenorhabditis elegans Meiosis by Antagonizing Inhibitory Effects of Synaptonemal Complex Precursors
Source: PLoS Genet. 2012 Aug 9;8(8):e1002880. doi: 10.1371/journal.pgen.1002880 (PMC3415444; doi:10.1371/journal.pgen.1002880)

**wt**

DNA

SUN-1  
S8-Pi

**chk-2**

DNA

SUN-1  
S8-Pi

**hal-2**

DNA

SUN-1  
S8-Pi

**hal-2**

DNA

SUN-1  
S8-Pi

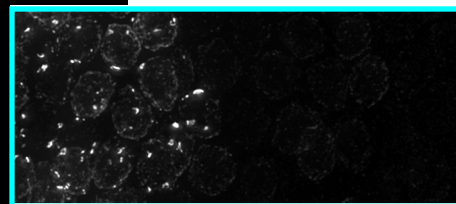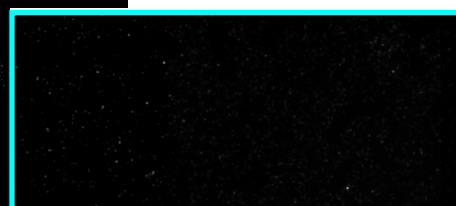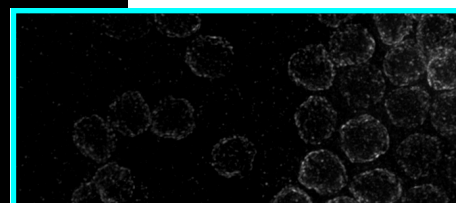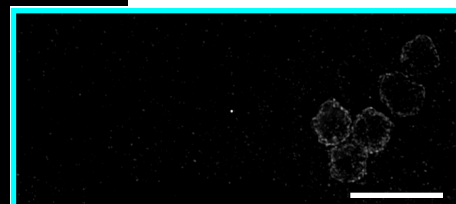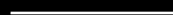

Supplement: Figure S1 — SUN-1 S8-Pi is severely reduced and/or delayed in hal-2 mutants. Immunolocalization of SUN-1 S8-Pi in DAPI-stained gonads of the indicated genotypes, with meiosis progressing from left to right. In the meiotic prophase region of the wild-type germ line, SUN-1 S8-Pi immunostaining is most robust from the TZ to mid-pachytene stage and concentrates as bright NE patches and also localizes weakly throughout the NE (as shown in zoomed-in image). In contrast, no SUN-1 S8-Pi is detected in the corresponding region of the chk-2 mutant germ line. Two representative examples of hal-2 mutant germ lines are shown; SUN-1 S8-Pi is not detected in the regions corresponding to the transition zone of wild-type germ lines, and exhibits only diffuse NE localization (as shown in zoomed-in images) in a subset of nuclei in the early and mid-pachytene regions. Bar, 50 µm. Bar in zoomed-in images, 10 µm. (PDF) [file pgen.1002880.s001.pdf]

*hal-2*

HIM-8 LMN-1

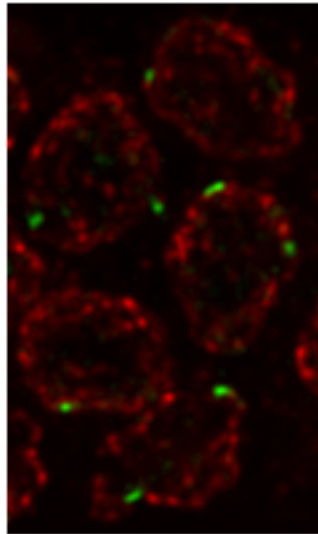

HIM-8 LMN-1  
DNA

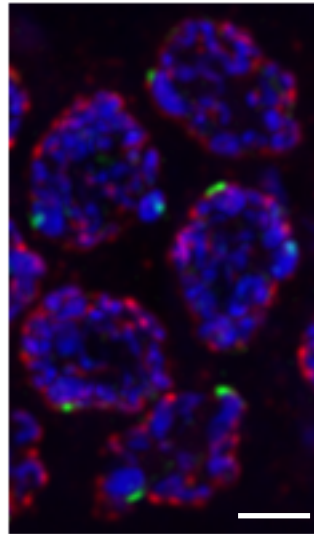

Supplement: Figure S2 — HIM-8 localizes close to the nuclear envelope in a hal-2 mutant. IF images of hal-2 early prophase nuclei co-stained with antibodies against HIM-8 and nuclear lamin LMN-1, which localizes immediately adjacent to the inner surface of the NE. Images shown are 3D projections of partial data stacks in which sections from the tops and bottoms of the nuclei were omitted in order to illustrate the close proximity of the HIM-8 foci to the NE; not all HIM-8 foci were contained within the projections shown. Bar, 2 µm. (PDF) [file pgen.1002880.s002.pdf]

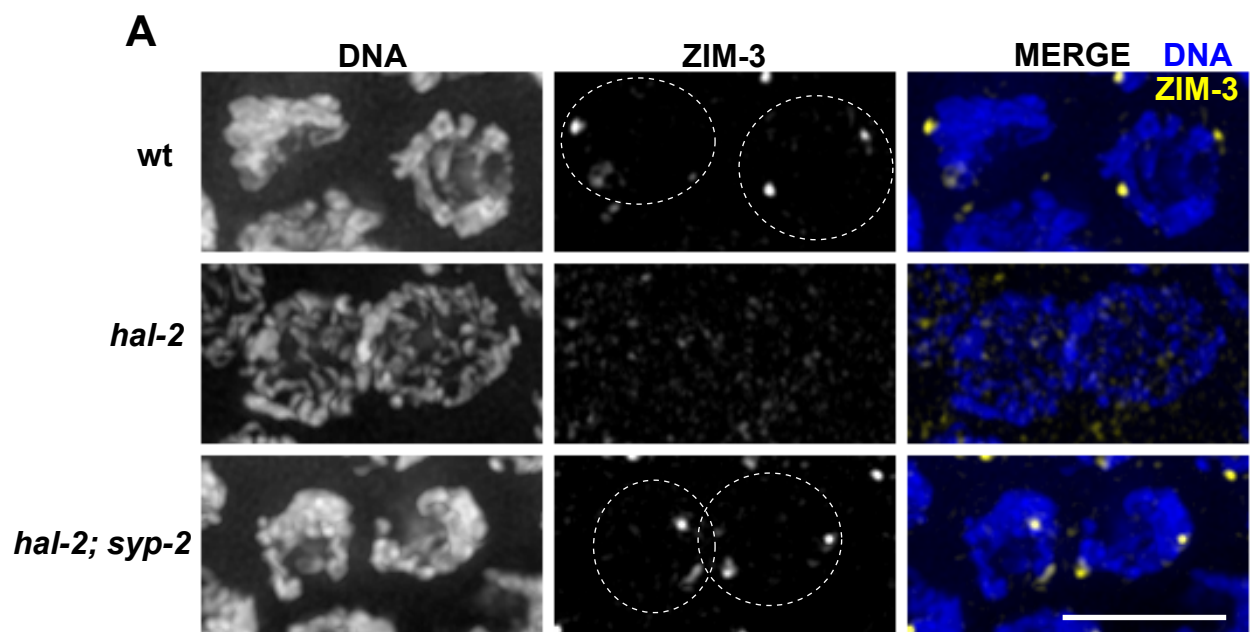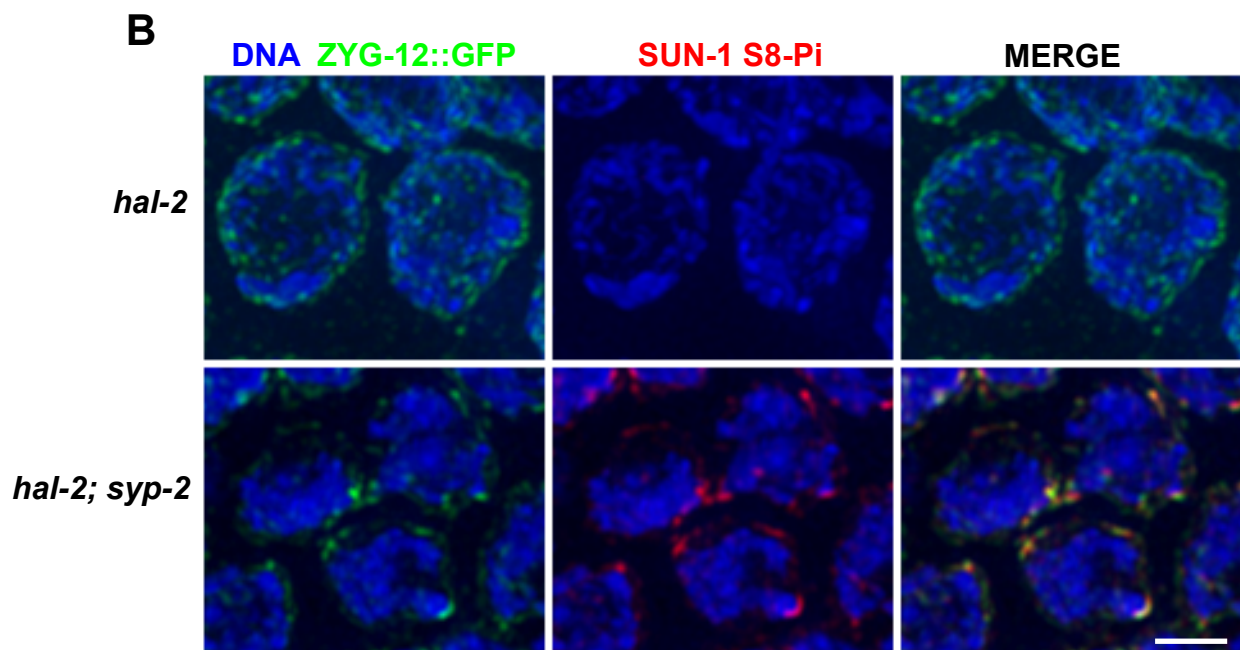

Supplement: Figure S3 — Bright ZIM-3 foci and NE patches of ZYG-12::GFP and SUN-1 S8-Pi are restored in hal-2; syp-2 double mutants. (A) IF images of early prophase nuclei stained with antibodies against ZIM-3, the PC-binding protein specific for chromosomes I and IV. Bright ZIM-3 foci are detected near the NE of wild-type TZ nuclei (top panel), whereas the hal-2 mutant nuclei lack clear ZIM-3 foci (middle panel). ZIM-3 foci are present in TZ nuclei of the hal-2; syp-2 double mutant (bottom panel), indicating that the concentration of autosomal PC-binding proteins to bright NE-associated foci was restored. Bar, 5 µm. Animals used for these IF images contained the zyg-12::gfp transgene. (B) IF images of early prophase nuclei stained for ZYG-12::GFP and SUN-1 S8-Pi in hal-2 and hal-2; syp-2 germ lines. Chromosome clustering, SUN-1 Ser8 phosphorylation and NE patches containing ZYG-12::GFP and SUN-1 S8-Pi (features that are missing in the hal-2 nuclei) are restored in the hal-2; syp-2 nuclei. Bar, 2 µm. Animals used for these IF images contained the zyg-12::gfp transgene. (PDF) [file pgen.1002880.s003.pdf]

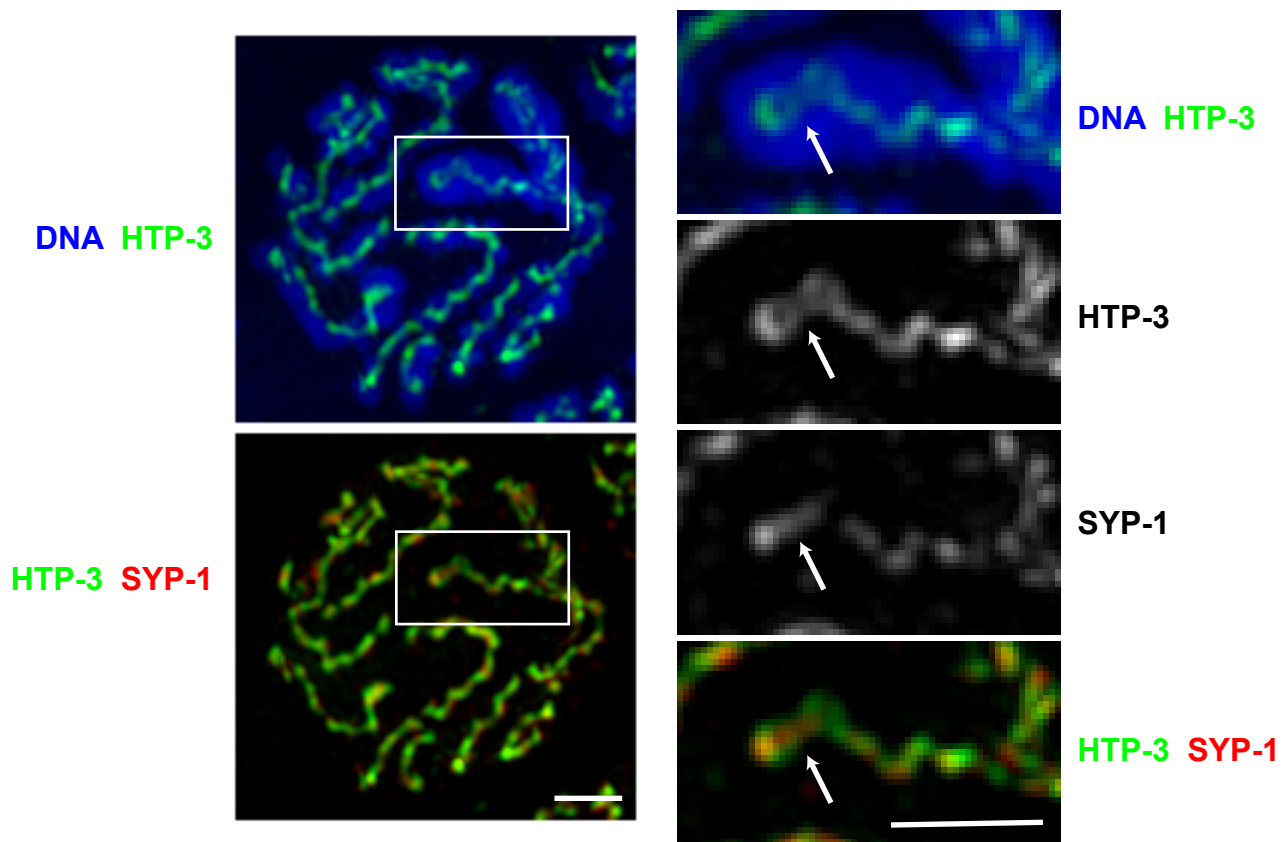

Supplement: Figure S4 — Regions of fold-back near chromosome ends observed in hal-2 mutants. 3D-SIM images of a hal-2 mutant pachytene nucleus stained for the LE component HTP-3 and CR protein SYP-1. A short stretch of parallel LEs flanking a single track of SYP-1 (as shown by right panel of zoomed-in images of region in white box) is detected in the hal-2 mutant nucleus and corresponds to a region of fold-back near the end of the chromosome. Images shown are 3D projections of partial data stacks in which the bottom half of the nucleus was excluded in order to highlight the region of fold-back. Bars, 1 µm. (PDF) [file pgen.1002880.s004.pdf]

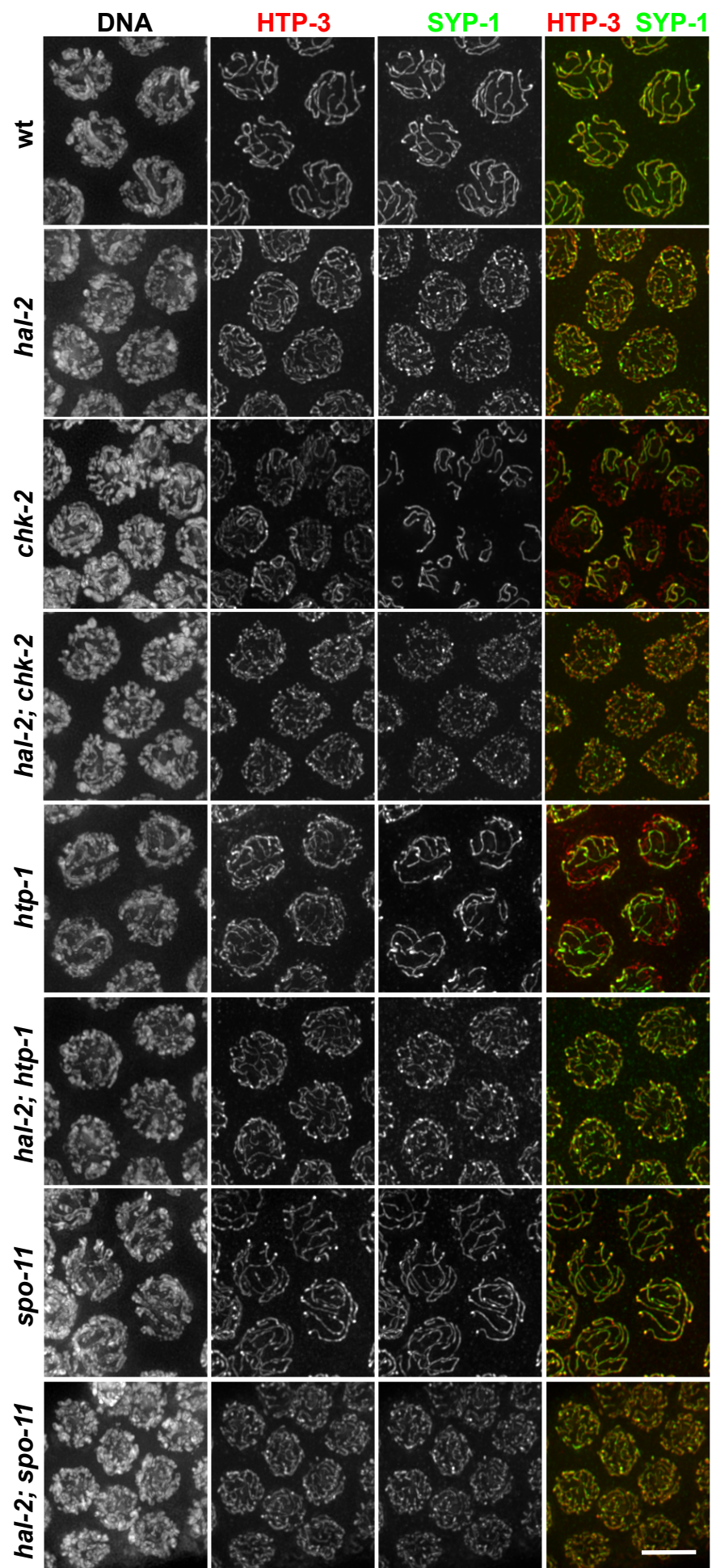

Supplement: Figure S5 — SYP-1 localization in hal-2; chk-2 , hal-2; htp-1 and hal-2; spo-11 double mutants resembles that in hal-2 mutants. IF images of HTP-3 and SYP-1 in late pachytene nuclei of the indicated genotypes. Images show: 1) many asynapsed chromosomes (HTP-3 stretches lacking SYP-1) and limited SYP-1 localized at the interface between nonhomologous chromosome segments in the chk-2 mutant; 2) extensive synapsis (between paired X chromosomes and between nonhomologous chromosomes) in the htp-1 mutant; 3) full homologous synapsis in the spo-11 mutant; 4) SYP-1 localization in hal-2; chk-2, hal-2; htp-1 and hal-2; spo-11 double mutants, which appears similar to that observed in the hal-2 single mutant, in that extensive SYP-1 is observed to be colocalized with HTP-3 along the axes of unpaired homologs. Bar, 5 µm. (PDF) [file pgen.1002880.s005.pdf]

DNA SUN-1 S8-Pi

*syp-3; hal-2*

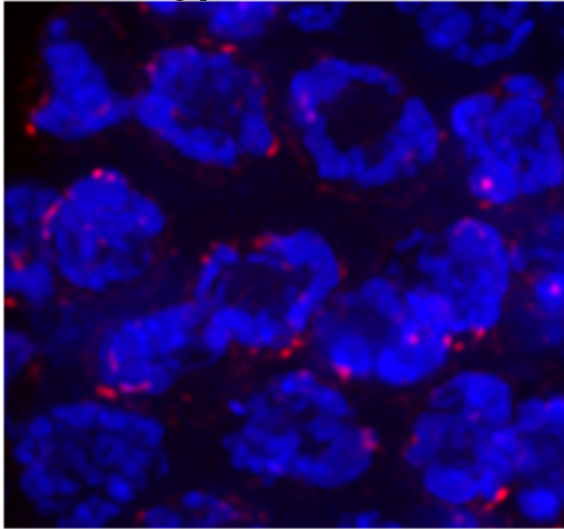

*syp-3; hal-2; chk-2*

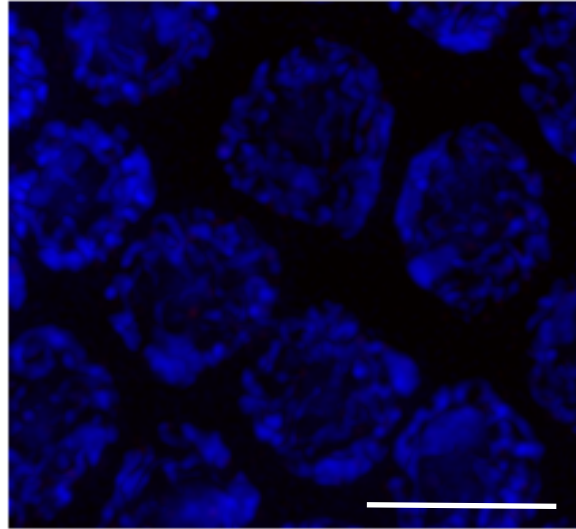

Supplement: Figure S6 — Markers of PC-mediated chromosomal movement in syp-3; hal-2 mutants are dependent on chk-2 . IF images of early prophase nuclei stained with antibodies against SUN-1 S8-Pi reveal the presence of TZ nuclei with clustered chromosomes and SUN-1 S8-Pi NE patches in a syp-3; hal-2 mutant (left panel) and the lack of these features in syp-3; hal-2; chk-2 triple mutant nuclei (right panel), indicating that these markers of PC-mediated chromosomal movement are dependent on chk-2. Bar, 5 µm. (PDF) [file pgen.1002880.s006.pdf]

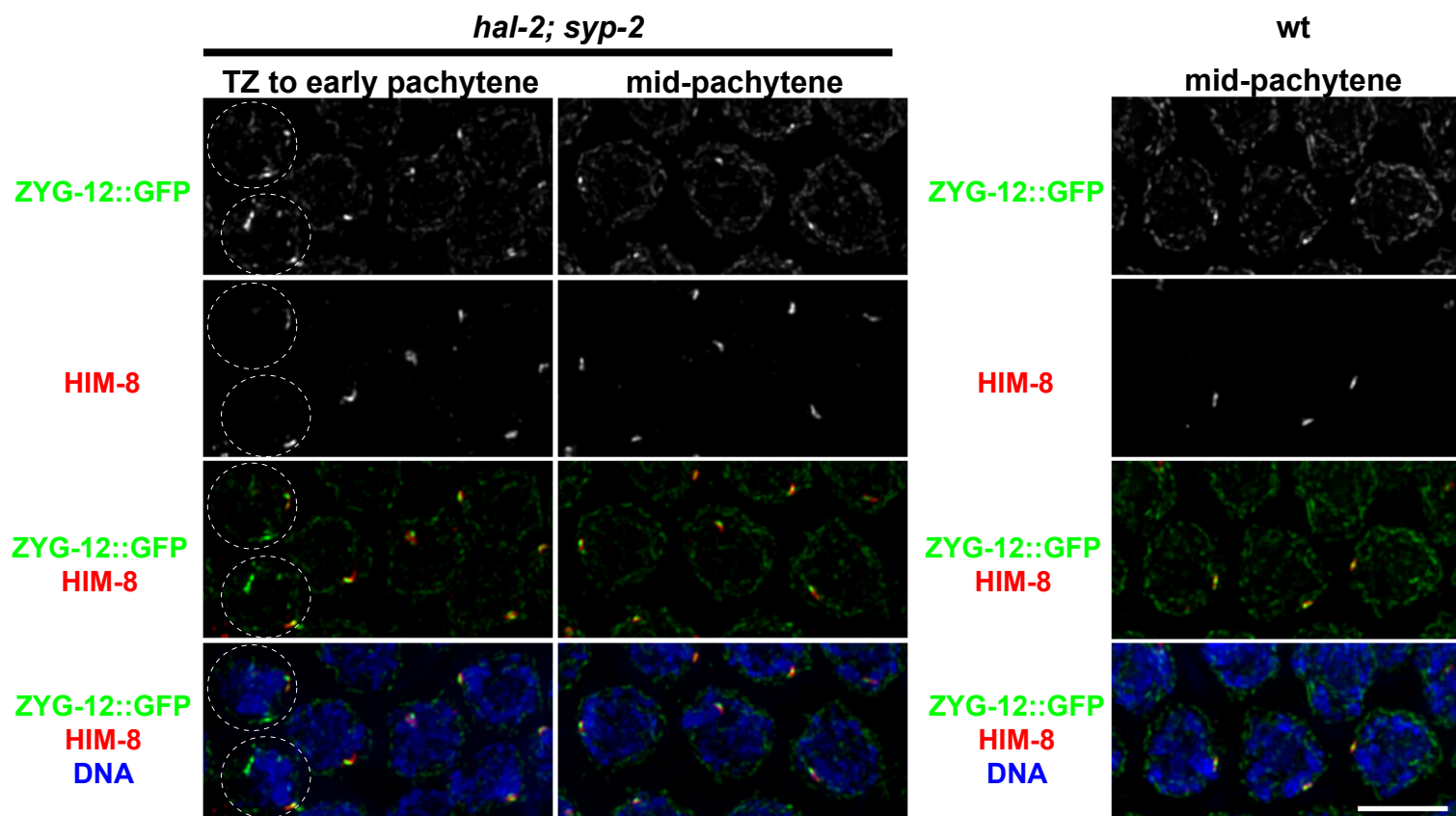

Supplement: Figure S7 — A single ZYG-12::GFP focus associated with HIM-8 persists into pachytene of hal-2; syp-2 mutants. IF images of wild-type and hal-2; syp-2 mutant nuclei of the indicated meiotic stages, co-stained for ZYG-12::GFP and HIM-8. In wild-type germ lines, a single ZYG-12::GFP NE focus associated with HIM-8 persists in nuclei that have exited the clustered chromosome configuration (right panels). Likewise, hal-2; syp-2 double mutants exhibit TZ nuclei with multiple ZYG-12::GFP patches (circled nuclei in left panels), and after release from the clustered chromosome configuration, a single ZYG-12::GFP NE focus colocalizing with HIM-8 persists in pachytene nuclei (left and middle panels). Bar, 5 µm. Animals used for these IF images contained the zyg-12::gfp transgene. (PDF) [file pgen.1002880.s007.pdf]

*hal-2; him-3*

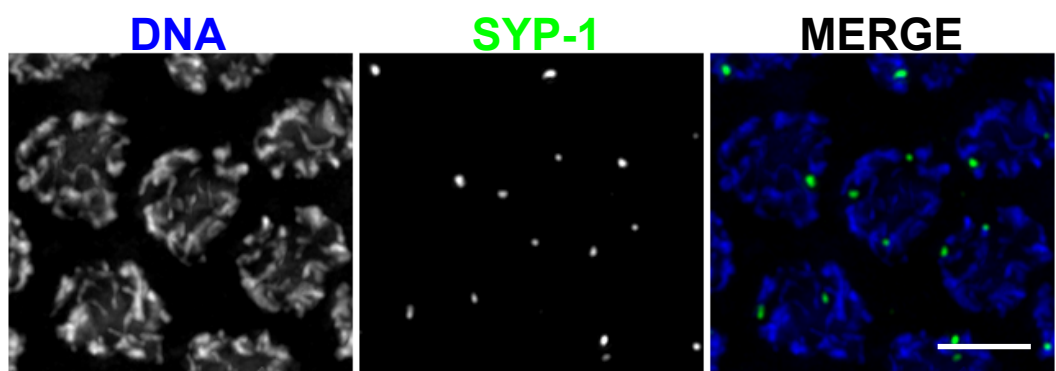

Supplement: Figure S9 — SYP-1 forms aggregates and does not localize on chromosomes in hal-2; him-3 double mutants. IF images of pachytene nuclei from a hal-2; him-3 double mutant, showing that SYP-1 is not loaded on chromosomes but does form nuclear aggregates, indicating that HAL-2 is not required for aggregate formation. Bar, 4 µm. (PDF) [file pgen.1002880.s009.pdf]

SUN-1 S8-Pi

wt

transition zone

*hal-2*

no transition zone

*syp-2*

transition zone

*hal-2; syp-2*

transition zone

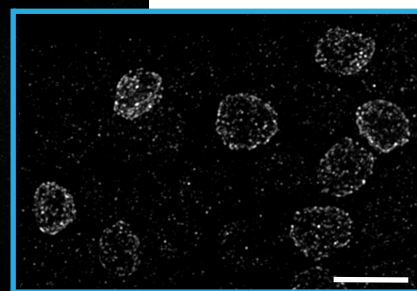

Supplement: Figure S10 — Diffuse SUN-1 S8-Pi NE localization in hal-2 mutant gonad shown in Figure 8A . Immunolocalization of SUN-1 S8-Pi in gonads of the indicated genotypes, with meiosis progressing from left to right. Images shown are the same gonads as those shown in Figure 8A. In the hal-2 mutant gonad shown, early meiotic prophase nuclei lack SUN-1 S8-Pi, and only a small subset of nuclei at a later stage of meiotic prophase exhibit diffuse SUN-1 S8-Pi NE signal, as demonstrated in the zoomed-in image (blue box). The SUN-1 S8-Pi signal was boosted in the zoomed-in image to illustrate the weak diffuse NE localization. Bars, 10 µm. (PDF) [file pgen.1002880.s010.pdf]

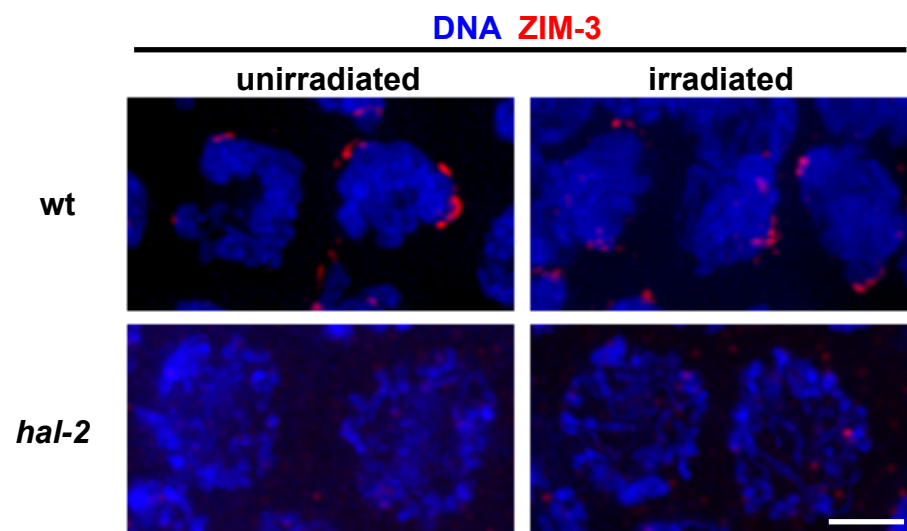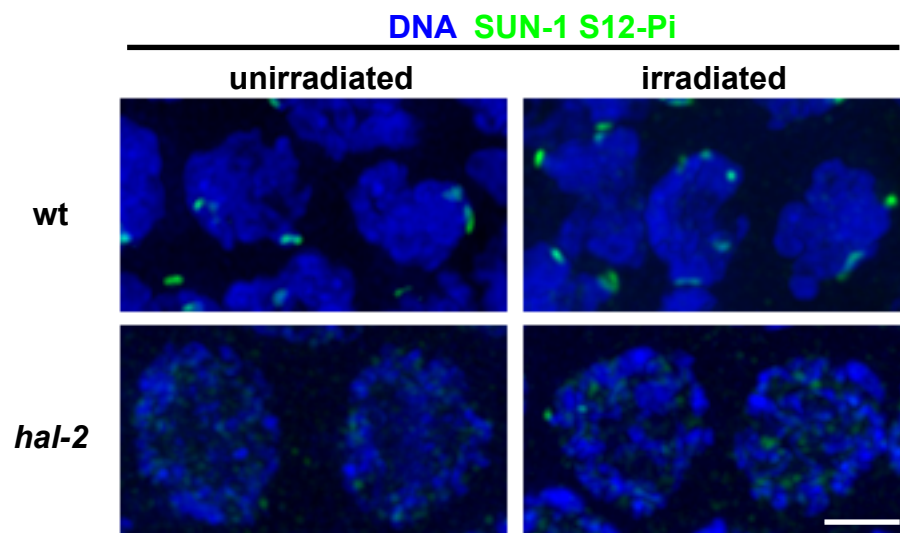

Supplement: Figure S11 — Irradiation-induced DSBs do not restore chromosome clustering, bright NE-associated ZIM-3 foci or NE patches of SUN-1 S12-Pi in hal-2 mutant gonads. IF images of wild-type and hal-2 mutant early prophase nuclei stained for SUN-1 S12-Pi and ZIM-3. As in unirradiated hal-2 controls, chromosome clustering, ZIM-3 recruitment to PCs and the nuclear envelope, and formation of SUN-1 S12-Pi NE patches are not observed in hal-2 gonads exposed to 5 krad of γ irradiation. Bar, 2 µm. (PDF) [file pgen.1002880.s011.pdf]
