# Supplementary material for: HAL-2 Promotes Homologous Pairing during Caenorhabditis elegans Meiosis by Antagonizing Inhibitory Effects of Synaptonemal Complex Precursors
Source: PLoS Genet. 2012 Aug 9;8(8):e1002880. doi: 10.1371/journal.pgen.1002880 (PMC3415444; doi:10.1371/journal.pgen.1002880)
Supplement: Figure S8 — Modified gene prediction for hal-2/T16H12.11. (A) Modified predicted coding sequence for the hal-2/T16H12.11 gene is shown in capital letters, with the additional coding sequence highlighted in yellow; upstream untranslated sequence is shown in lower case. (B) Alignment of the N-terminal portions of HAL-2 orthologs from five different Caenorhabditis species, showing strong conservation of the first N-terminal 13 amino acids. Red indicates identity among all five orthologs; blue indicates conservative substitutions; green indicates semi-conservative substitutions. (PDF) [file pgen.1002880.s008.pdf]

A

```
>T16H12.11 corrected coding sequence
gtcaattttgtacaatttagATGAACACACCGGTAAAAGCAAAACGGCAAAGGCAGCAAATGCCAGGAA
CTCCGATACCCATGGGTCGGCAAACGCACAGGGCACATTCCGCCTCTGGTTTCTACACTCCTCACGTCTC
AAAATTGAAGACAAACTATGACAGAGAGTTTCAAAAGTTTCTTCGATCTGCCAAAACCGCAATCGATCAC
TTGGATAAGATTGAAATGCAAATACTTTGCCCGCTGAAAAAGAAGATAAAACGTTTTCACAAGGAATCCA
ACACTCTCGATGTCTACTTCAGGTCACAAACATATTGGAGATCCTTCTACAAAGAGCTTATGAACCGCAG
CGATGATGTCATGGATTGCTTTCGATTCGAGGATCTCCGTGACGTTGCTGCAGTTGTTGAGAGTCATCGA
TTGCCGGATGTGTTGGTTTCATTTGTCACGCAGCAGTTGATGGAATGGAGAGAATTGTTCAAAGACGTCG
AGTGGATGCGAAAGTTTCGTGAATTGAGCCACAATATGGATTCTTTGAAAACGAAAAGAATTGAGCATGT
GCTGCGACAAGACATCATCAGGGAGATTGGTGTAGCCATCGATGTGCACAATCTGAAAATCGATGATTTC
AGAAGTCACAGCAATAACACGGAACGGGTCGCGGAAGGAATTGCTCATGCCATTGAAGAGTCCAAGAATA
AACAAGATGCTGGGAATCAGTTGAAGCATGTTCCTGTGTTATCCAAGCAGATGAGTGTTCGACGATGGTA
TCGAGAACACTATGGTGACCCGCAAGCTTTGTCAGATGAATCAGATGAAGAATCCTTGTCTGATGGTGAC
AATTCGTCTCTTTTTGTTGGCACGGCAGATTCAAGCAGTCGTGACGCTAGTGACATATCGACGGAAGAAG
TGGTCACCGAAAACGAATTCGACGAATTCTTCAATTAG
```

B

C. elegans	MNT-PVKAKRQR-QQMPGTPIPMGRQTHRAHSASGFYTPHVSKLKTNYDREFQKFLRSAK
C. briggsae	MIT-PEKPKRSRLQQQPGTPIPTSRRRDDLVNIPAVYNPPVSKLKKNYDLEFGRFLRSAK
C. remanei	MST-PVQVKRSR-NQQPGTPIPSSRRREPSSLTAAVYNPPVSKLKKNYDQEFDRFLRSAK
C. brenneri	MNT-PVKVKKSR-QNQGGTPIPATRRRHIASPVETVYNPPVLKLKKNYDVEFERFLQSAK
C. japonica	MNTSPAKVKRAR-QEHLDTPIPAPSRKQRKMSLMGVYNPSVTRLKRTYSLEFGKFLKSLK
