# Supplementary material for: HAL-2 Promotes Homologous Pairing during Caenorhabditis elegans Meiosis by Antagonizing Inhibitory Effects of Synaptonemal Complex Precursors
Source: PLoS Genet. 2012 Aug 9;8(8):e1002880. doi: 10.1371/journal.pgen.1002880 (PMC3415444; doi:10.1371/journal.pgen.1002880)
Supplement: Table S1 — High embryonic lethality and Him phenotype in hal-2 mutants are rescued by GFP::HAL-2. (DOC) [file pgen.1002880.s012.doc]

**Table S1**

**High embryonic lethality and Him phenotype in *hal-2* mutants are rescued by GFP::HAL-2**

| Genotype | Average Number of Embryos  (Number of Broods) | Total Number of Embryos | Percent Dead Eggs | Percent Males |
| --- | --- | --- | --- | --- |
| *hal-2* | 67 + 16 (17) | 1147 | 87.2 | 32.7 |
| *gfp::hal-2* | 196 + 48 (10) | 1962 | 0.4 | 0.1 |
| *gfp::hal-2; hal-2* | 99 + 18 (10) | 993 | 1.6 | 0.5 |
