# Supplementary material for: HAL-2 Promotes Homologous Pairing during Caenorhabditis elegans Meiosis by Antagonizing Inhibitory Effects of Synaptonemal Complex Precursors
Source: PLoS Genet. 2012 Aug 9;8(8):e1002880. doi: 10.1371/journal.pgen.1002880 (PMC3415444; doi:10.1371/journal.pgen.1002880)
Supplement: Text S1 — Supplemental materials and methods. (PDF) [file pgen.1002880.s013.doc]

Text S1

Supplemental Materials and Methods

Genetics

The following strains were used in this study:

AV 327	*hal-2(me79)/qC1[qls26] III*
AV 146	*chk-2(me64) rol-9(sc148)/unc-51(e369) rol-9(sc148) V*
AV 276	*+/nT1[unc-?(n754)let-?] IV; syp-2(ok307)/nT1 V*
AV 393	*htp-1(gk174)/nT1[unc-?(n754)let-?qIs50] (IV;V)*
AV 521	*+/nT1 IV; scc-3(ku263)/nT1[qIs51] V*
AV 562	*+/nT1[unc-?(n754)let-?qIs50] IV; scc-3(ku263) syp-1(me17)/nT1 V*
AV 568	*meIs9[unc-119(+) pie-1*^{promoter}*::gfp::syp-3]; unc-119(ed3) III*
AV 597	*hal-2(me79)/qC1[qls26] III; him-3(gk149)/nT1[qIs51] IV; +/nT1 V*
AV 616	*hal-2(me79)/qC1[qls26] III; ojIs9*
AV 617	*hal-2(me79)/qC1[qls26] III; +/nT1[qIs51] IV; syp-2(ok307)/nT1 V*
AV 644	*+/nT1 IV; syp-2(ok307)/nT1[qIs51] V; ojIs9*
AV 646	*hal-2(me79)/qC1[qls26] III; +/nT1[qIs51] IV; syp-2(ok307)/nT1 V; ojIs9*
AV 659	*hal-2(me79) III; meIs10*
AV 663	*hal-2(me79)/qC1[qIs26] III; meIs9*
AV 665	*hal-2(me79)/qC1[qIs26] III; spo-11(ok79)/nT1 IV; +/nT1[qIs51] V*
AV 684	*+/hT2[qIs48] I; hal-2(me79)/hT2 III*
AV 690	*syp-3(ok758)/hT2[qIs48] I; hal-2(me79)/hT2 III*
AV 691	*+/hT2[qIs48] I; hal-2(me79)/hT2 III; chk-2(me64) rol-9(sc148)/+ V*
AV 698	*syp-3(ok758)/hT2[qIs48] I; hal-2(me79)/hT2 III;*

	chk-2(me64) rol-9(sc148)/+ V
AV 706	*meIs10[unc-119(+)pie-1*^{promoter}*::gfp::hal-2]; unc-119(ed3) III*
AV 711	*+/hT2[qIs48] I; hal-2(tm4960)/hT2 III*
AV 724	*hal-2(me79)/qC1[qls26] III; htp-1(gk174)/nT1[qIs51] IV; +/nT1 V*
CV 2	*syp-3(ok758)/hT2[bli-4(e937)let-?(q782)qIs48] I; +/hT2 III*
TY 4986	*htp-3(y428)ccIs4251 I/hT2[bli-4(e937)let-?(q782)qIs48] (I;III)*
VC 418	*him-3(gk149)/nT1[qIs51] IV; +/nT1 V*
WH 223	*ojIs9 [zyg-12ABC::gfp unc-119(+)]; unc-119(ed3) III*

Another deletion, *tm1767*, was reported to have a 675 bp deletion and a 9 bp insertion in *T16H12.11*. However *tm1767* worms did not display any meiotic defects, and we detected full length HAL-2 protein in *tm1767* animals by Western blotting, indicating that there is a functional copy of *hal-2* in this strain.

Transgenic Strain Construction

Transgenic strains (AV568 and AV706) were generated by microparticle bombardment [1]. For the expression of an N-terminal GFP::SYP-3 fusion protein driven by the *pie-1* promoter, a construct was made by cloning all 1065 bp of the *syp-3* gene and its predicted 3'UTR into the SpeI site of pIC26 [2], which contains an *unc-119(+)* rescuing fragment, and the construct was used for bombardment into *unc-119(ed3)* animals. The transgenic strain expressing N-terminal GFP::HAL-2 was generated similarly to the *gfp::syp-3* strain except that the bombardment construct was built by inserting a 867 bp *hal-2* cDNA (missing the first 60 bp of the coding sequence) into the SpeI site of pIC26 [2].

RNAi

Double-stranded RNA (dsRNA) was made as in [3]. Two different sets of primers were used to generate *hal-2* dsRNA, and both sets elicited similar RNAi phenotypes: 1st primer set is 5'-TAA TAC GAC TCA CTA TAT GCC AAC AAA AAG AGA C -3' and 5'-TAA TAC GAC TCA CTA TAA CAC CGG TAA AAG CAA A -3'; 2nd primer set is 5'-TAA TAC GAC TCA CTA TAC GAA CAC TCA TCT GCT T -3' and 5'- TAA TAC GAC TCA CTA TAC ACT TTT CGG ACG GGT T -3'. RNAi was performed as described in [3].

Cytological Analysis

DeltaVision images were acquired as 3D stacks of 0.2 μm optical sections using a 60x oil objective with 1.5x optivar, and except where noted, images shown are full projections through 3D data stacks comprising of whole nuclei, generated with a maximum-intensity algorithm.

Antibodies used for IF: The following primary antibodies were used at the indicated dilutions: guinea pig anti-HIM-8 (1:500) [4], rabbit anti-HIM-3 (1:200) [5], guinea pig anti-SYP-1 (1:200) [6], goat anti-SYP-1 (for 3D-SIM images, 1:1000) [7], rabbit anti-HTP-1/2 (1:500) [8], rabbit anti-RAD-51 (1:500) ([9]; affinity purified by A. Tam), rabbit anti-GFP (1:1000) (Villeneuve lab), chicken anti-GFP (1:2000) (Abcam 13970), chicken anti-HTP-3 (1:250 or 1:500) [10], guinea pig anti-ZIM-2 (1:2000) [11], rabbit anti-ZIM-3 (1:5000 or 1:1000) [11], guinea pig anti-SUN-1 S8-Pi (1:700 or 1:1000) [12], rat anti-LMN-1 (1:500) [13], rabbit anti-PLK-2 (1:5) [14], guinea pig anti-SUN-1 S12-Pi (1:1500) [12] and rabbit anti-HAL-2 (1:10000) (this study). Affinity-purified HAL-2 antibody was generated by SDI (Newark, DE), using the final 100 amino acids as the

antigen. For DeltaVision images, all secondary antibodies were Alexa Fluor goat (1:400) from Invitrogen. For 3D-SIM images, commercial secondary antibodies conjugated to DyLight 488, 594, and 649 (Jackson Immunoresearch) were used at 1:500 dilution.

FISH: FISH was performed similarly to that in [15,16] with the following modifications: Worms were dissected in egg buffer with 1% Tween-20 and fixed in 2% paraformaldehyde (PFA) for 1 min. Fixed samples were then frozen in liquid nitrogen and placed into 95% ethanol at -20˚C for 1 min. Next, the samples were fixed in 4% PFA for 10 min at room temperature and then washed in 2X SSCT (2X SSC with 0.1% Tween-20). The slides were then successively incubated in 2X SSCT containing increasing concentrations of formamide (5% to 50%). After that, the samples were incubated in 2X SSCT with 50% formamide at 37˚C for 2 hours. A hybridization solution with the labeled probes was then added to the slides and the slides were subsequently processed on an OmniSlide flat bed thermal cycler (Thermo) with a 10 min heat denaturation of 80˚C, followed by stepwise cooling to 37˚C in 4 min and hybridization at 37˚C overnight. Slides were then washed for 30 min twice in 2X SSCT with 50% formamide at 37˚C. After the washes, the slides were successively incubated in 2X SSCT with decreasing formamide concentrations until a final 2X SSCT wash. Then the samples were placed in 2X SSCT with DAPI for 10 min and washed in 2X SSCT, followed by another 2X SSC wash before being mounted in Vectashield (Vector Laboratories).

Quantitative Pairing Analysis of 5S rDNA, HIM-8 and ZIM-2

Quantitative analysis of pairing was carried as in [17] with minor modifications. Briefly, the length of each gonad (from the distal tip to end of pachytene) was measured and the gonads were then divided into 5 regions of equal lengths. For nuclei that were completely

contained within the data stacks, distances between peak intensities of signals (5S rDNA FISH, HIM-8 or ZIM-2) were measured with the IVE software package [18] and the signals were considered paired if the distance between them was ≤ 0.7 μm. At least 3 gonads of each genotype were quantified. For 5S FISH, the total number of nuclei scored in each zone was as follows (Zone 1 to Zone 5): wild type: 183, 143, 184, 166 and 104. *hal-2*: 91, 113, 170, 159 and 91. *syp-3; hal-2*: 131, 179, 184, 183 and 69. *syp-3*: 142, 170, 195, 135 and 61. The animals used in the pairing analyses of HIM-8 and ZIM-2 were all carrying the *zyg-12::gfp* transgene. For HIM-8, the total number of nuclei scored in each zone was as follows (Zone 2 to Zone 5): wild type: 136, 156, 130 and 67. *hal-2*: 95, 112, 80 and 39. *hal-2; syp-2*: 96, 131, 150 and 84. *syp-2*: 164, 164, 132 and 81. For ZIM-2, the total number of nuclei scored in Zone 2 was as follows: wild type: 279. *hal-2:* 157. *hal-2; syp-2*: 120. *syp-2*: 223. Fisher's Exact Test was performed for statistical analyses of pairing data using InStat 3 software (http://www.Graphpad.com).

Western Blot Analysis

Worms were collected and washed in M9 before being suspended in SDS-PAGE sample buffer (4 worms per μl). Worm suspension was then boiled and separated on a 4-15% SDS-PAGE gradient gel (50 worms per lane). Western analysis was performed using standard procedures with rabbit anti-HAL-2 (1:10000) and mouse anti-α-TUBULIN (1:10000; Sigma; clone DM1A).

References

1. Praitis V, Casey E, Collar D, Austin J (2001) Creation of low-copy integrated transgenic lines in *Caenorhabditis elegans*. Genetics 157: 1217-1226.

2. Cheeseman IM, Niessen S, Anderson S, Hyndman F, Yates JR, 3rd, et al. (2004) A conserved protein network controls assembly of the outer kinetochore and its ability to sustain tension. Genes Dev 18: 2255-2268.

3. Colaiacovo MP, Stanfield GM, Reddy KC, Reinke V, Kim SK, et al. (2002) A targeted RNAi screen for genes involved in chromosome morphogenesis and nuclear organization in the *Caenorhabditis elegans* germline. Genetics 162: 113-128.

4. Phillips CM, Wong C, Bhalla N, Carlton PM, Weiser P, et al. (2005) HIM-8 binds to the X chromosome pairing center and mediates chromosome-specific meiotic synapsis. Cell 123: 1051-1063.

5. Zetka MC, Kawasaki I, Strome S, Muller F (1999) Synapsis and chiasma formation in *Caenorhabditis elegans* require HIM-3, a meiotic chromosome core component that functions in chromosome segregation. Genes Dev 13: 2258-2270.

6. MacQueen AJ, Colaiacovo MP, McDonald K, Villeneuve AM (2002) Synapsis-dependent and -independent mechanisms stabilize homolog pairing during meiotic prophase in *C. elegans*. Genes Dev 16: 2428-2442.

7. Harper NC, Rillo R, Jover-Gil S, Assaf ZJ, Bhalla N, et al. (2011) Pairing centers recruit a Polo-like kinase to orchestrate meiotic chromosome dynamics in *C. elegans*. Dev Cell 21: 934-947.

8. Martinez-Perez E, Schvarzstein M, Barroso C, Lightfoot J, Dernburg AF, et al. (2008) Crossovers trigger a remodeling of meiotic chromosome axis composition that is linked to two-step loss of sister chromatid cohesion. Genes Dev 22: 2886-2901.

9. Colaiacovo MP, MacQueen AJ, Martinez-Perez E, McDonald K, Adamo A, et al. (2003) Synaptonemal complex assembly in *C. elegans* is dispensable for loading

strand-exchange proteins but critical for proper completion of recombination. Dev Cell 5: 463-474.

10. MacQueen AJ, Phillips CM, Bhalla N, Weiser P, Villeneuve AM, et al. (2005) Chromosome sites play dual roles to establish homologous synapsis during meiosis in *C. elegans*. Cell 123: 1037-1050.

11. Phillips CM, Dernburg AF (2006) A family of zinc-finger proteins is required for chromosome-specific pairing and synapsis during meiosis in *C. elegans*. Dev Cell 11: 817-829.

12. Penkner AM, Fridkin A, Gloggnitzer J, Baudrimont A, Machacek T, et al. (2009) Meiotic chromosome homology search involves modifications of the nuclear envelope protein Matefin/SUN-1. Cell 139: 920-933.

13. Liu J, Rolef Ben-Shahar T, Riemer D, Treinin M, Spann P, et al. (2000) Essential roles for *Caenorhabditis elegans* lamin gene in nuclear organization, cell cycle progression, and spatial organization of nuclear pore complexes. Mol Biol Cell 11: 3937-3947.

14. Labella S, Woglar A, Jantsch V, Zetka M (2011) Polo kinases establish links between meiotic chromosomes and cytoskeletal forces essential for homolog pairing. Dev Cell 21: 948-958.

15. Dernburg AF, McDonald K, Moulder G, Barstead R, Dresser M, et al. (1998) Meiotic recombination in *C. elegans* initiates by a conserved mechanism and is dispensable for homologous chromosome synapsis. Cell 94: 387-398.

16. Nabeshima K, Mlynarczyk-Evans S, Villeneuve AM (2011) Chromosome painting reveals asynaptic full alignment of homologs and HIM-8-dependent remodeling

of X chromosome territories during *Caenorhabditis elegans* meiosis. PLoS Genet 7: e1002231.

17. MacQueen AJ, Villeneuve AM (2001) Nuclear reorganization and homologous chromosome pairing during meiotic prophase require *C. elegans chk-2*. Genes Dev 15: 1674-1687.

18. Chen H, Hughes DD, Chan TA, Sedat JW, Agard DA (1996) IVE (Image Visualization Environment): a software platform for all three-dimensional microscopy applications. J Struct Biol 116: 56-60.
